# Emotion Induction Modulates Neural Dynamics Related to the Originality of Ideational Creativity

**DOI:** 10.1002/hbm.70182

**Published:** 2025-03-12

**Authors:** Radwa Khalil, Sascha Frühholz, Ben Godde

**Affiliations:** ^1^ School of Business, Social and Decision Sciences Constructor University Bremen Germany; ^2^ Cognitive and Affective Neuroscience Unit Zurich Switzerland; ^3^ Department of Psychology University of Oslo Oslo Norway

**Keywords:** creativity, cross‐frequency coupling, emotion, exploitation, exploration, functional connectivity, happy, ideational originality, neutral, sad

## Abstract

Emotions remarkably impact our creative minds; nevertheless, a comprehensive mapping of their underlying neural mechanisms remains elusive. Therefore, we examined the influence of emotion induction on ideational originality and its associated neural dynamics. Participants were randomly presented with three short videos with sad, neutral, and happy content. After each video, ideational originality was evaluated using the alternate uses task. Both happy and sad inductions significantly enhanced ideational originality relative to the neutral induction condition. However, no significant difference was observed in ideational originality between the happy and sad emotion inductions. Associated neural dynamics were assessed through EEG time‐frequency (TF) power and phase‐amplitude coupling (PAC) analyses. Our findings suggest that emotional states elicit distinct TF and PAC profiles associated with ideational originality. Relative to baseline, gamma activity was enhanced after the neutral induction and more enhanced after the induction of a happy emotion but reduced after the induction of sad emotion 2–4 s after starting the task. Our functional connectivity couplings suggest that inducing happy and sad emotions may influence the working memory and attentional system differently, leading to varying effects on associated processing modes. Inducing a happy emotion may result in decreased neural activity and processing of rich information in working memory for exploring more original ideas through cognitive flexibility. In contrast, inducing a sad emotion may enhance neural activity and increase coupling within the attention system to exploit and select fewer original ideas through cognitive persistence.

## Graphical abstract

Summary of the effects of mood induction on ideational originality and associated neural dynamics.

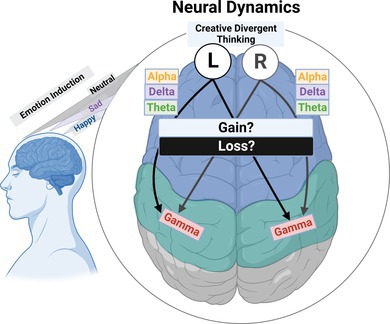

**Abbreviations**
AUT: alternate uses task
CFC: cross‐frequency coupling
DA: dopamine
DN: default network
DT: divergent thinking
EC: executive control
EEG: electroencephalography
fMRI: functional magnetic resonance imaging
PAC: phase‐amplitude coupling
PFC: prefrontal cortex
SAM: Self‐Assessment Manikin
sEBR: spontaneous eye blinking
SemDis: computing semantic distance
TF: time frequency
WM: working memory

**Summary**

- Ideational originality was significantly higher during happy and sad induction than in neutral conditions.
- Emotional induction elicited distinct EEG time‐frequency and phase‐amplitude coupling profiles associated with ideational originality.
- Relative to baseline, gamma activity was enhanced in the neutral induction and more robust in a happy induction but reduced in a sad state 2–4 s after starting AUT.

## 1 Introduction

Creative thinking is a dynamic process that involves shifting between multiple modes of thought to generate novel and useful ideas (Dietrich and Kanso 2010; Girn et al. 2020; Kleinmintz et al. 2019; Runco and Jaeger 2012; Sowden et al. 2015; Zamani et al. 2021). It is widely accepted that emotional states considerably influence creativity (Baas et al. 2008; Davis 2009; Khalil et al. 2019; Khalil, Lin, et al. 2023; Leung et al. 2021; Lin et al. 2014; Mastria et al. 2019; Tsai et al. 2013; Zenasni and Lubart 2002). The influence of emotional states on creativity, mainly whether positive or negative moods facilitate or inhibit creativity, is a topic of ongoing debate in the literature (Baas et al. 2008; Davis 2009; Khalil et al. 2019). This debate underscores the complexity of the relationship between emotions and creative thinking. Based on the *hedonic‐tone hypothesis* (Davis 2009) and *the activating hypothesis* (Amabile 1983; de Dreu et al. 2008; Simonton 1997), emotional states can either facilitate or hinder creative thinking because they are more activating than neutral conditions.

Previous studies indicate that positive emotions can enhance creativity, while negative emotions impede original ideation (Baas 2015; Baas et al. 2008; de Dreu et al. 2008; Mastria et al. 2019). The dual‐pathway model to creativity model (Nijstad et al. 2010) encapsulates this contrasting effect, suggesting that while activating positive emotions promotes cognitive flexibility and exploration, activating negative emotions can enhance persistence and focused processing (Baas et al. 2008; de Dreu et al. 2008). De Dreu et al. (2008) asserted that fear and anger are expected to improve persistence rather than cognitive flexibility in the dual pathway model of creativity. In contrast, deactivated emotions like sadness may foster creativity under certain conditions. Furthermore, research indicates that music with negative emotional tones can enhance originality in creative tasks (Xiao et al. 2023), challenging the idea that only positive emotions promote creativity. Psychological research evaluates emotions through two main dimensions: valence and activation. Valence refers to the intrinsic nature of an emotional state—whether it is positive (attractive) or negative (aversive) (Russell 2003). Activation, on the other hand, indicates the level of energy or arousal associated with that emotion. This dimensional approach is widely accepted in emotional research, as it provides a more nuanced understanding of how emotions influence cognitive processes and behavior (Posner et al. 2005). This model conceptualizes this concept by positioning emotions in a two‐dimensional space defined by valence and arousal. For example, joy and excitement exhibit high positive valence and high activation, while sadness and lethargy correspond to low valence and low activation. Emotions can also occupy intermediate positions, creating a spectrum of emotional experiences that can vary in their impact on cognitive functioning (Russell 2003).

The increase in the level of original ideation after experiencing happiness implies that individuals tend to feel more joyful and playful in a positive mood than in a negative one (Hirt et al. 1997). Previous research has identified a connection between dopamine (DA) and original ideation (Agnoli et al. 2023; Chermahini and Hommel 2010, 2012; de Rooij and Vromans 2020; Ueda et al. 2016). DA, often associated with a positive mood, catalyzes creative thinking (Baas et al. 2008; Davis 2009; Khalil et al. 2019). Several studies investigating the correlation between originality and DA activity indicated by the rate of spontaneous eye blinking (sEBR) (Agnoli et al. 2023; Chermahini and Hommel 2010, 2012; de Rooij and Vromans 2020; Ueda et al. 2016). Therefore, providing an empirical integrative framework for evaluating the neural mechanisms underlying the emotional and cognitive neural elements, which is still pending, should be systematically addressed (Dietrich 2004; Dietrich and Kanso 2010; Khalil et al. 2019).

One of the most common operationalizations of creative cognition is divergent thinking (DT), which signifies the cognitive process of generating multiple, varied ideas or solutions to open‐ended problems (Guilford 1950). Tests of DT are one of the most frequently used assessments of creative potential. The alternate uses task (AUT; Guilford 1967) is among the most commonly used assessment methods for measuring DT. In the AUT, participants are requested to list alternative uses for everyday objects, such as bricks, clips, toothbrushes, newspapers, and so forth. Several theories in creativity research have emphasized the originality in DT as a crucial component of creativity and effectiveness for an idea to be considered creative (Agnoli et al. 2023; Corazza 2016; Rothenberg and Hausman 1976; Runco 2008; Runco et al. 2005; Runco and Jaeger 2012). The subjective‐based scoring method is commonly used to evaluate the originality of idea generation. This methodological approach uses subjective criteria rather than objective metrics to assess the originality of responses based on descriptive judgments from human evaluators, often guided by specific subjective criteria.

There is growing evidence that the neural mechanisms of creative ideation are not solely reliant on a single mental process or specific brain region, such as asymmetric hemispheric activation (Martindale et al. 1984), alpha synchronization (Fink et al. 2009), low arousal (Martindale 1999) or defocused attention (Mendelsohn 1976). The process of transforming novel ideas into creative behavior in the prefrontal cortex (PFC) and its brain circuits involves evaluating the original idea generation (Dietrich 2004; Gonen‐Yaacovi et al. 2013; Huang et al. 2018; Khalil, Agnoli, et al. 2023; Khalil et al. 2019, 2020; Khalil and Demarin 2024; Khalil and Moustafa 2022; Kleinmintz et al. 2019; Mok 2014). Information is processed by PFC circuits, which dynamically activate other neural circuits such as sensory cortices, emotional limbic regions, motivational systems, and sensorimotor‐related regions for processing visual, auditory, affective, and aversive information (Dietrich 2004; Huang et al. 2018; Khalil et al. 2019; Khalil and Demarin 2024; Khalil and Moustafa 2022; Kleinmintz et al. 2019; Mok 2014).

Studies examining brain dynamics and functional connectivity of creative thinking utilize electroencephalography (EEG)^1^ (Agnoli et al. 2018; Dietrich and Kanso 2010; Fink and Benedek 2014; Schwab et al. 2014; Srinivasan 2007) and functional magnetic resonance imaging (fMRI) (Beaty et al. 2015; Beaty et al. 2016, 2019, Beaty and Johnson 2021; Beaty et al. 2018; Fink et al. 2010; Huang et al. 2018; Zamani et al. 2021). Research using EEG to study creative thinking consistently reveals two neural features. The first implies an elevation in task‐related alpha‐power in frontal brain areas during creative tasks compared to baseline (Agnoli et al. 2018; Benedek et al. 2011; Fink et al. 2007; Fink et al. 2009; Rominger et al. 2019; Schwab et al. 2014). The second indicates a hemispheric asymmetry with a right‐hemispheric dominance for alpha power (Aghababyan et al. 2007; Bhattacharya and Petsche 2005; Corazza et al. 2022; Fink et al. 2009; Grabner et al. 2007, 2018; Martindale et al. 1984; O'Rourke et al. 2015; Srinivasan 2007).

Creative thinking relies on a balance of spontaneous thought processes and controlled top‐down activity (Abraham 2016; Beaty et al. 2016). The extensive research in this area has firmly established the executive nature of creativity (Benedek et al. 2014, 2023; Benedek and Fink 2019; Benedek and Neubauer 2013; Khalil, Agnoli, et al. 2023; Khalil et al. 2019, 2020; Khalil, Lin, et al. 2023; Khalil and Demarin 2024; Khalil and Moustafa 2022); for review, see Palmiero et al. (2022). Moreover, it was suggested that functional connectivity between the frontal and parietal cortices implies the involvement of attention during creative ideation (Beaty et al. 2016, 2019; Beaty et al. 2018; Gonen‐Yaacovi et al. 2013; Rominger et al. 2019). It was reported that pianists who expressed emotions increased functional connectivity between the dorsolateral PFC and the default network (DN) during improvisation (Pinho et al. 2016). The DN is notable for its activation in the resting state. It is mainly involved in self‐generated thought, which can be spontaneous (i.e., mind‐wandering) or goal‐directed (i.e., mental navigation) (Beaty et al. 2016; Khalil and Demarin 2024; Khalil and Moustafa 2022). The frontoparietal network and executive control (EC) are involved in high‐level cognitive processes, including decision‐making, creative ideation, problem‐solving, attention, working memory (WM), and regulating thoughts and actions (Beaty and Johnson 2021; Lara and Wallis 2014; Ptak 2012). Interestingly, it was suggested that the two systems (EC and DN) have opposite effects on attention. Frontoparietal activity directs attention to external stimuli (Ptak 2012), while DN activity internally directs cognition (Andrews‐Hanna 2012). WM function is partly supported by frontoparietal activity as part of the EC network (Palva et al. 2010; Pessoa et al. 2002). Thus, other potential EEG biomarkers of creative thinking could be identified by utilizing neural network analysis. These biomarkers may capture the dynamics of the two key neural features of creative thinking mentioned earlier.

In recent years, phase‐amplitude coupling (PAC) has become a central analytical approach in EEG research, providing valuable insights into the interactions between different frequency bands that support brain function (Canolty and Knight 2010). PAC constitutes a specific form of cross‐frequency coupling (CFC), which examines the relationship between the phase of low‐frequency oscillations and the amplitude of high‐frequency oscillations. CFC describes how neural oscillations across distinct frequency bands interact within and between various brain regions, resulting in a complex regulatory framework (Canolty and Knight 2010). For example, CFC, when associated with subnetworks of WM between parietal low‐frequency and frontal high‐frequency bands, indicates facilitating the maintenance and guidance of information over a short period (Roux and Uhlhaas 2014). This analysis elucidates how neural circuits integrate and transfer information across different temporal and spatial scales (for a comprehensive overview, see Senkowski and Engel (2024)). PAC measures the oscillatory coupling between the low‐frequency brain rhythm phase and high‐frequency component amplitude (Senkowski and Engel 2024). Hence, it coordinates neural activity (Canolty et al. 2006; Canolty and Knight 2010) and enhances information transfer efficiency (Tort et al. 2010). This coupling is associated with cognitive and neural mechanisms, including executive functions such as WM, attention, and information integration processing across brain regions (Canolty and Knight 2010; Daume et al. 2017; Dimitriadis et al. 2015; Gwon et al. 2021; Szczepanski et al. 2014; Vaz et al. 2017). Therefore, PAC allows integration activity across various spatial and temporal scales, ensuring coordinated information processing across different brain regions (Canolty and Knight 2010; Gwon et al. 2021; Tort et al. 2010). Low‐frequency oscillations, particularly in the theta and alpha ranges, frequently synchronize distant brain areas; simultaneously, high‐frequency gamma activity is associated with local computations such as sensory processing or memory encoding (Roux and Uhlhaas 2014; Solomon et al. 2017; Voytek et al. 2010; Zioga et al. 2024).

For these reasons, functional connectivity analyses^2^ that utilize PAC and CFC have the advantage of illustrating how local computations—represented by high‐frequency activity—are influenced by broader, network‐level communications, indicated by low‐frequency oscillations during creative ideation. Previous research identified a creative ideation process through PAC involving theta, alpha, and gamma frequency bands (Marmpena et al. 2016); other research examined the fronto‐parietal coupling patterns that were associated with increasing creative ideation (Bose et al. 2019). These patterns could be interpreted as modulating the interaction among connector nodes within the fronto‐parietal network and their corresponding systems, elucidating the functional connectivity of integrated cognition (Bertolero et al. 2015, 2017, 2018; Murphy et al. 2020). Hence, PAC between frontal and parietal cortices might underlie attention networks, allowing the frontal cortex to exert top‐down control over parietal areas and enhancing task‐relevant sensory processing while suppressing irrelevant information.

Our study examines the influence of inducing emotion on ideational originality and the underlying neural network dynamics. Positive, neutral, and negative emotions were induced by introducing participants to three short emotional videos in counterbalanced order, each eliciting a different emotion (happy, neutral, and sad). Then, we evaluated ideational originality using AUT (Guilford 1967). The uniqueness of responses was assessed by employing the subjective scoring method (Beketayev and Runco 2016; Runco 2008). EEG was recorded during the AUT, and the associated neural network dynamics were evaluated using TF power and functional connectivity (i.e., PAC and CFC) analyses. We selected these two analyses to determine how the frontal and parietal areas communicate during original ideation. Our study was partially exploratory, as no previous research had examined the functional connectivity of creative ideation following the induction of emotional states. We anticipated the following outcomes: (1) variations in the scoring of ideational originality between emotional conditions; (2) distinctive neural TF and fronto‐parietal PAC associated with the three emotional induction conditions during original ideation.

## 2 Methods

### 2.1 Participants, Ethical Approval and Eligibility Criteria

The study followed the Declaration of Helsinki (DoH) in all aspects. Participants were recruited from the student population of Constructor University, Germany. This recruitment was done through flyers and emails distributed within the university community. Flyers were placed strategically in high‐traffic areas, such as student lounges, libraries, and academic departments, to maximize visibility and attract potential participants. These flyers provided essential information about the study, including its purpose, eligibility criteria, and time commitment required. A QR code was also included to facilitate easy access to the online registration form. In parallel, emails were sent to various departments and student organizations, inviting students to participate in the study—this multi‐faceted approach aimed to create a diverse participant pool, enhancing the sample's representativeness. To further encourage participation, students who completed the study were offered incentives, such as entry into a raffle for gift cards or extra credit in their courses. This strategy increased participation rates and fostered a sense of appreciation for the time and effort contributed by participants.

Eligibility criteria were set to focus the study on a particular demographic. Participants were required to be undergraduate students at the university, aged between 18 and 30, and proficient in English. These criteria aimed to minimize confounding variables associated with age, educational background, and language skills. Before the study, participants completed a questionnaire about their mental and physical health, drug and medication use, and family history of disease before the experiment. Those with a history of diagnosed neurological disease or psychiatric disorders, heart conditions, severe head injuries, seizures (personally or in first‐degree relatives), recurring syncopes, or learning disabilities were excluded.

Once the participants expressed interest and met the eligibility criteria, they received detailed information about the study's procedures, including informed consent forms. All participants underwent screening to determine their eligibility for the EEG procedure and signed an informed consent form before beginning the experiment. None of the participants had any prior experience with the AUT. The experiment was conducted in a controlled environment to minimize distractions and ensure session consistency. The sample consisted of 28 (14 male and 14 female) healthy undergraduate students between 18 and 23 years of age. Two participants were excluded from the EEG analysis because their recordings did not have clear EEG event markers. As a result, this study's final sample was 26 (13 male and 13 female).

### 2.2 Experimental Procedure

**FIGURE 1:**
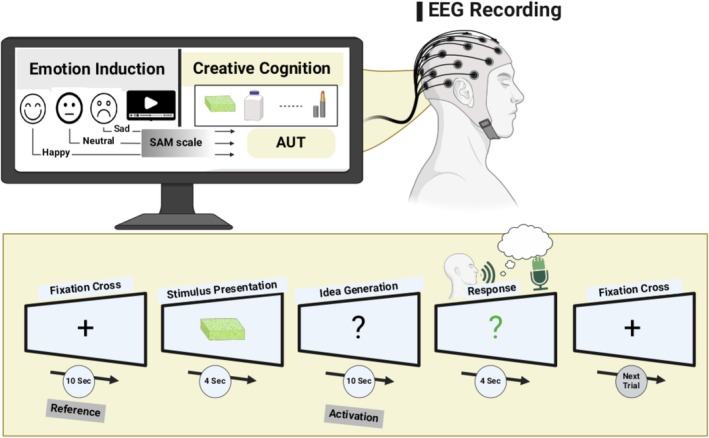
Diagram of the experimental setup. Participants were introduced to three short videos with different emotional contents—sad, neutral, and happy—for 5 min each in counterbalanced order. After each video, the participants were given the Self‐Assessment Manikin (SAM) scale to rate their emotional arousal and pleasure levels and check the effectiveness of emotion induction. The creative ideation was evaluated using the alternative uses task (AUT). The idea generation process was instructed to generate *original, untraditional uses* of stimulus presentation. The EEG was recorded during the AUT for time‐frequency (TF) and functional connectivity analysis.

Participants were introduced to three short videos with three emotional contents—sad, neutral, and happy—for 5 min each in a counterbalanced order, Figure 1.

Each film clip lasted 5 min, followed by a 2‐min interval for participants to complete a brief Self‐Assessment Manikin (SAM) questionnaire (Bradley and Lang 1994). The order of film clips was counterbalanced across participants to control for order effects. A total of three possible orders were created, and participants were randomly assigned to one of these orders to ensure that each emotional condition was presented in a balanced manner. After each video, participants were asked to rate the SAM scale to evaluate their emotional arousal and pleasure levels, assessing the effectiveness of the emotion induction. Next, their ideational originality scores were evaluated using AUT. EEG was recorded during the AUT to analyze the associated time frequency (TF) and functional connectivity (i.e., PAC and CFC) with the original ideation.

### 2.3 Emotion Induction

Before starting the AUT, emotional states were induced through short film clips that lasted for 5 min each. These film clips were adapted from Tsai et al. (2013) and cover a broad spectrum of emotional states. They were selected from standardized emotion‐eliciting film sets (Gross and Levenson 1995; Schaefer et al. 2010), each designed to evoke a specific emotional response. These sets include Comedic or uplifting scenes, such as *Funny Cats* videos, to elicit happiness, joy, or amusement. Clips, such as *Big Cat Diary*, are often used to evoke neutral emotional states, designed to elicit low emotional intensity and minimal affect. Films that evoke sadness, such as *The Champ* (famous for its sadness‐inducing scenes), are commonly used to evoke negative emotions, such as sadness.

After each film, participants rated their current emotional states of arousal and pleasure using the 5‐point SAM scale (Bradley and Lang 1994). The SAM is a well‐validated, nonverbal pictorial assessment technique that directly measures the arousal, pleasure, and dominance associated with a participant's affective reaction to various stimuli. The SAM scale was used to ensure the effectiveness of the emotion induction. The subjects rated their arousal and pleasure after watching each short film. The most positive effect was coded with the number 5, whereas the most negative effect was coded with the number 1. It ranged from a smiling, happy figure to a frowning, unhappy figure.

### 2.4 Alternative Uses Task

In the AUT, everyday objects, such as a sponge or bottle, were presented on the screen, and participants were asked to generate unconventional, original uses for these objects. Examples of these items are illustrated in the Appendix. Participants were carefully instructed on performing the AUT following the conventional task instructions (Runco et al. 2010; Runco and Acar 2012). The AUT's presentation started with a fixation cross for 10 s (reference period; Schwab et al. (2014)). Following the fixation, the stimulus picture of an everyday object appeared on the screen for 4 s. This stimulus was followed by a white question mark instructing participants to generate one *original use* of the presented object within 10 s. Subsequently, the color of the question mark changed to green, signaling that the participants could articulate their ideas within the next 4 s. The oral responses of participants were recorded and transcribed before the subsequent trial started. Fifteen items adapted from the experimental protocol by Schwab et al. (2014) were applied in each condition of emotion induction, that is, neutral, happy, and sad. The presentation of the AUT stimuli was fully randomized, and we assured each participant that there was no repeated item to avoid any memory rumination of the items. We used the standard AUT subjective scoring method from the Runco Creativity Assessment Battery (Beketayev and Runco 2016; Runco and Jaeger 2012).

### 2.5 Subjective Originality Measures

Two experienced raters were involved in the evaluation process to assess the subjective originality score. Subjective scoring methods effectively reflect original ideation by complementing objective measures of creative ideation (Silvia et al. 2008). To mitigate any potential rating bias, we initially transcribed each recorded participant's responses into a spreadsheet and organized them alphabetically within each category. The raters then independently scored the responses on a scale from 1 (not at all original) to 5 (highly original), which was recommended as a scoring criterion by Wilson et al. (1953) for capturing individual differences in originality. Interrater reliability was adequate (Cohen's *κ* = 0.87).

We used automated scoring to strengthen confidence in our subjective originality measures and provide a more robust originality analysis. These automated methods offer consistent and objective evaluations, minimizing potential biases associated with human raters (Beaty et al. 2022; Beaty and Johnson 2021). For instance, methods such as computing semantic distance (SemDis; Beaty et al. 2022; Beaty and Johnson 2021) have strongly correlated with traditional subjective assessments, confirming the reliability of the originality estimates derived from AUT. Accordingly, we calculated SemDis scores for the originality of responses generated in the AUT and evaluated their correlation coefficient strength between SemDis and the subjective scoring assigned by the raters.

### 2.6 EEG Recording and Offline Processing

EEG was measured simultaneously with the AUT. A 32‐channel EEG was recorded using Ag‐AgCl electrodes mounted on an elastic cap according to the 10–20 electrode system, with a reference electrode at the nose tip (Nz). The EEG signal was amplified by a REFA multi‐channel system (TMS International; www.tmsi.com) and digitized at a sampling rate of 512 Hz. Impedances were kept below 10 kΩ. A horizontal and vertical electrooculogram (EOG) was recorded at the outer canthi of the eyes and the supra‐ and suborbital positions of the right eye to monitor eye blinks and movements.

We used EEGLAB software (version 2019.1; https://sccn.ucsd.edu/eeglab/index.php) for offline preprocessing and TF decomposition of the EEG data. The EEG signal was then re‐referenced to an average across the 32 EEG electrodes and downsampled to a 256 Hz sampling rate during offline preprocessing. The signals from channels with large signal artifacts and signal drifts were reconstructed using the spherical interpolation method from neighboring electrodes (one electrode for six participants and two electrodes for one participant). We applied filtering to the EEG signal by first applying a notch filter to account for the 50 Hz direct current effects (47–53 Hz). Afterward, we applied a bandpass filter in the 0.1–100 Hz range. Blink‐artifact detection and correction were performed using a spatially independent component analysis (ICA; infomax algorithm) implemented in the EEGLAB software. The EEG data were epoched around references and activation period onsets in a broad prestimulus and poststimulus time range (−8 to +18 s).

TF analysis was applied to identify relevant frequency bands and their temporal changes during encoding (Maris and Oostenveld 2007). For this purpose, a continuous wavelet transformation was performed on single‐epoched trials for each subject. Four different clusters of electrodes were placed over the left frontal (F3, FC5, FC1), right frontal (F4, FC6, FC2), left parietal (P3, CP5, CP1), and right parietal (P4, CP6, CP2) areas. The TF procedure allows analyses of all oscillatory activity, phase‐locked and non‐phase‐locked, to the stimulus onset. The signal from each electrode and epoch was convolved with complex Morlet wavelets as follows: *w*(*t*, *f*0) = *A* exp(−*t*2/[2*σ*2*t*]) exp(2*iπf*0*t*), with *σf* = *1*/[2*πσt*]. The wavelets were normalized such that their total energy was 1. The central frequency *f*0 ranged between 1 and 70 Hz, with the ratio *f*0/*σf* = 7. Subsequently, the TF energy distribution of the reconstructed signal was obtained using Morlet wavelet expansion. The log‐scaled values obtained for each trial (each electrode cluster and each frequency) were normalized to the mean baseline energy level from −3000 to 0 ms before trial onset, revealing the percentage decrease or increase of the in‐band power during the encoding phase. The mean TF signal for the reference and activation periods was calculated separately for each electrode cluster and emotion condition for each participant.

The participants' mean TF signals were averaged across participants for each electrode cluster and emotion condition. The statistical significance of the relevant time and frequency areas was determined by a permutation test (2000 permutations), specifically by randomly shuffling time points and frequency bands for participants' mean TF signals within an electrode cluster, experimental period, and emotion condition. We calculated the difference in TF signals between the activation and the reference period for all permutations. We smoothed the TF signal by averaging over neighboring TF matrix elements (the center TF element and the first and second neighboring TF elements) to obtain a distribution of 2000 values for each TF signal of the four electrode clusters and three emotion conditions. The grand average and smoothed TF difference between the activation and the reference period were compared against this distribution of 2000 values using a normal cumulative distribution function. A time‐by‐frequency area was determined to be significant using a two‐sided test and *p* < 0.001. An additional cluster threshold was applied so that only TF areas with *k* ≥ 100 neighboring significant TF areas were determined to be significant. CFC analysis was performed between the two frontal electrode clusters and the two parietal electrode clusters. This CFC analysis was set up to determine the coupling between the 1–12 Hz range (delta: 1–3 Hz, θ: 3–7 Hz, alpha: 7–12 Hz) as the frequency phase range that convolved the amplitudes of the higher frequency in the low (30–50 Hz) and high (50–70 Hz) ranges.

The PAC patterns indicate the CFC between both hemispheres' frontal and parietal regions (hubs of the EC networks in creative ideation) during DT after emotion induction. PAC values represent the coupling strength between the phase of the low‐frequency oscillations (delta: 1–3 Hz, theta: 3–7 Hz, alpha: 7–12 Hz) in the frontal cortex and the amplitude of parietal's lower (30–50 Hz) and higher (50–70 Hz) gamma oscillations relative to all other factors that modulate the high‐frequency amplitude. Positive PAC values indicate a more robust modulation of parietal gamma by the phase of frontal low‐frequency oscillations (i.e., stronger coupling) during ideational originality than the baseline. On the contrary, negative PAC values indicate weaker modulation of parietal gamma oscillations by the phase of frontal low‐frequency oscillations (i.e., weaker coupling).

CFC analysis was computed using the Tensorpac toolbox (version 0.6.5; https://etiennecmb.github.io/tensorpac/) and the mean vector length method (Canolty et al. 2006), permuting the phase across trials (Tort et al. 2010) and normalizing data by subtracting and dividing utilizing the surrogates. All CFC analyses were performed separately for each participant, and the resulting CFC data was smoothed the same way as the TF data before we averaged them across participants. We used the same permutation procedure for the TF data to determine CFC areas of significance. A two‐sided test determined significance with *p* < 0.001 and *k* ≥ 12 for a significant number of neighboring areas.

### 2.7 Statistical Analysis

We analyzed all data using Jamovi software (R Core Team 2024; The Jamovi Project 2024). To test the effect of emotion induction on arousal and pleasure, we conducted one‐way ANOVAs with the mode of emotion induction (neutral, happy, and sad) as the within‐subjects factor and arousal and pleasure ratings as dependent variables, followed by post hoc comparison tests. A similar one‐way ANOVA was performed with ideational originality as the dependent variable, followed by a post hoc comparison test to test the effect of emotion induction on originality. Results were interpreted as statistically significant if the *p* value was < 0.05, corrected for multiple testing where applicable.

We performed Pearson correlational analyses between the PAC in each emotion induction condition to identify and characterize the specific patterns of PAC (i.e., connectivity patterns between frontal and parietal cortices) in each emotional induction condition. We then explored whether this correlation differs by gender. We conducted separate Pearson correlation analyses for male and female participants and compared the direction (i.e., positivity or negativity) and strength (i.e., significance) of these correlations. Data visualization was created using Graph Prism (GraphPad Software, San Diego, California USA, www.graphpad.com) and Adobe Illustrator (Adobe Inc 2019).

### 2.8 Ethical Approval

The authors state that this study was approved by the local ethics committee of Constructor University and has followed the principles outlined in the Declaration of Helsinki for human experimental investigations. Informed consent has been obtained from all the participants.

## 3 Results

### 3.1 Emotion Induction

**FIGURE 2:**
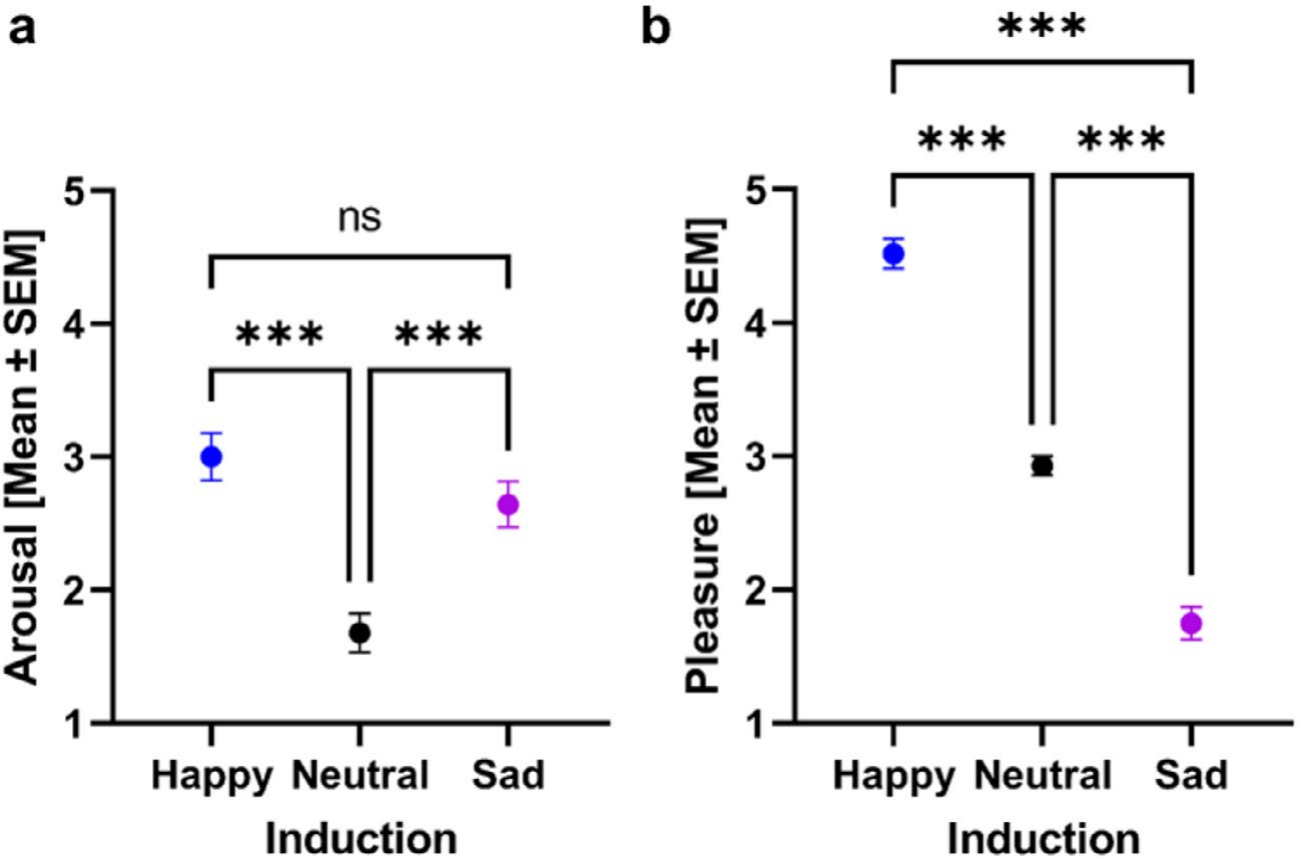
Effectiveness of emotion induction elicited through short films with happy, neutral, and sad emotional content. Panels (a) and (b) represent the effects on arousal and pleasure. The *y*‐axes of each panel refer to the mean and standard error of the mean [SEM] for arousal and pleasure after each emotion induction, as represented on the *x*‐axes. Stars indicate significance (*p < 0.05, **p < 0.01, ***p < 0.001).

Analysis of variance using general linear modeling revealed the effectiveness of emotion induction through videos with emotional content. ANOVA with the mode of emotion induction (neutral, happy, and sad) as the within‐subjects factor and arousal as the dependent variable revealed a significant main effect of induction mode (*F* (2,80) = 17.1, *p* < 0.001, *p η* ^2^ = 0.30; Figure 2a).

The mode of emotion induction through videos with emotional content affected arousal and pleasure differently. The highest level of arousal was observed in the happy induction mode; in contrast, the lowest level was observed in the neutral induction mode (Figure 2a). For pleasure, the highest level was found for the happy induction mode, while the lowest level was found for the sad induction mode (Figure 2b). Post hoc analysis confirmed significant differences between neutral and happy emotion induction modes (*t* = −5.63, *p*_Tukey_ < 0.001, df = 80) and between neutral and sad emotion induction modes (*t* = −4.15, *p*_Tukey_ < 0.001, df = 80). However, there were no significant differences between the happy and sad emotion induction modes (*t* = 1.52, *p*_Tukey_ = 0.29, df = 80).

Similarly, ANOVA with the mode of emotion induction as the within‐subjects factor and *pleasure* as the dependent variable revealed a significant main effect of induction mode (*F* (2,80) = 178, *p* < 0.001, *η* ^2^ = 0.82; Figure 2b). The highest level of pleasure was for the happy induction mode, while the lowest was for the sad induction mode. Post hoc analysis confirmed a significant difference between neutral and happy induction modes (*t* = −10.80, *p*_Tukey_ < 0.001, df = 80), neutral and sad induction modes (*t =* 8.08, *p*_Tukey_ < 0.001, df = 80), and happy and sad induction modes (*t* = 18.80, *p*_Tukey_ < 0.001, df = 80).

### 3.2 Effect of Emotion Induction on Ideational Originality

**FIGURE 3:**
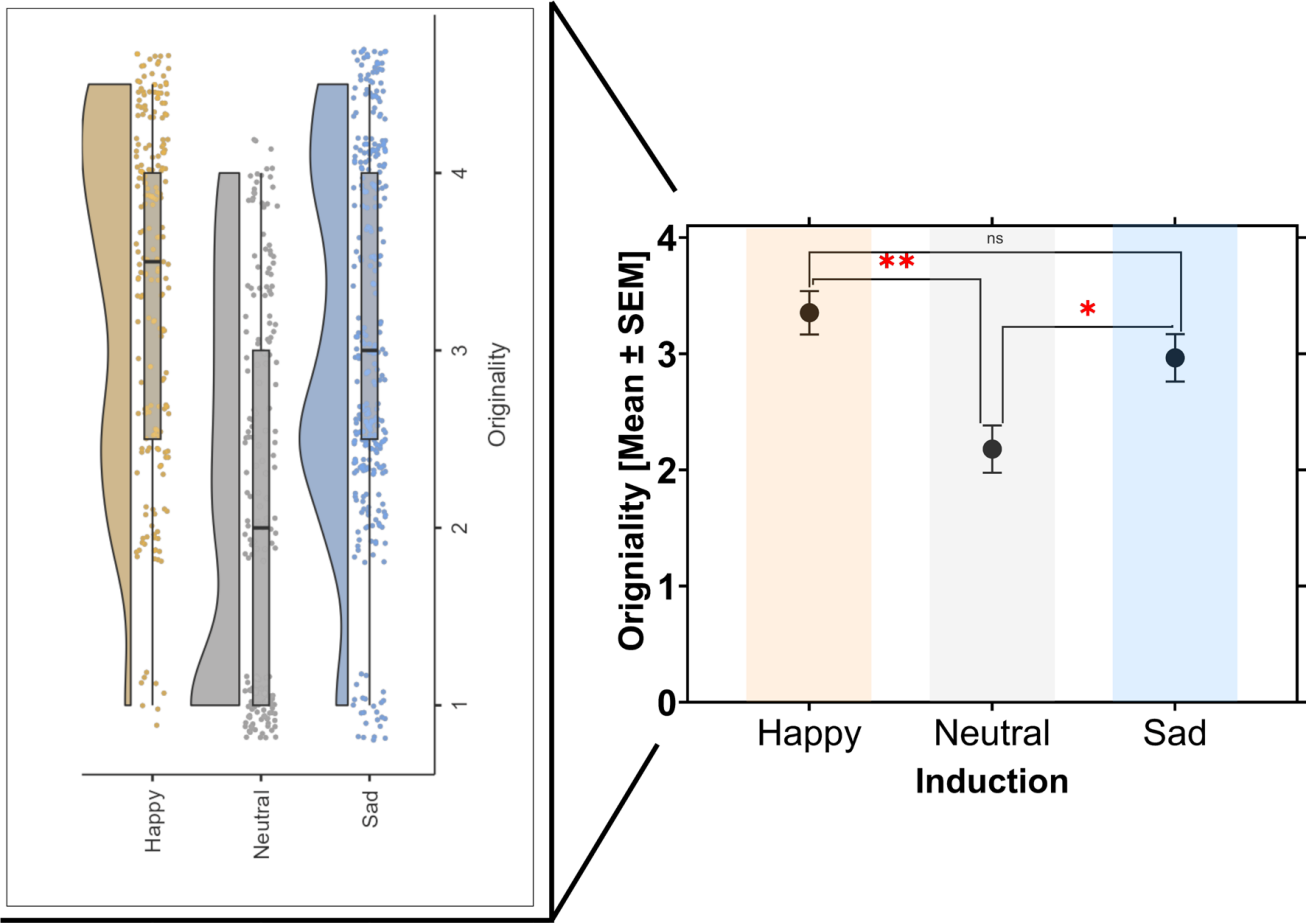
Effect of emotion induction on originality index. Emotion induction (happy, neutral, and sad) affects originality. The graph on the left side illustrates the differential frequency distribution of originality scores across the three emotional induction conditions. In the graph on the right, the y‐axes refer to the mean and standard error of the mean [SEM] for the originality scores after each emotion induction as represented on the x‐axes. Stars indicate significance (*p < 0.05, **p < 0.01, ***p < 0.001).

The effects of emotion induction on originality are illustrated in Figure 3.

ANOVA revealed a significant effect of emotional induction mode (*F* (2,80) = 9.87, *p* < 0.001, *η* ^2^ = 0.184). In contrast to the neutral condition, originality was enhanced in the happy and sad conditions. Post hoc tests confirmed significant differences in the originality scores between the neutral and happy induction modes (*t* = −4.16, *p*_Tukey_ < 0.001, df = 80) and a significant difference between the neutral and sad (*t* = −2.81, *p*_Tukey_ = 0.017, df = 80). However, there was no significant difference between the happy and sad induction modes (*t* = 1.37, *p*_Tukey_ = 0.359, df = 80). We used subjective scores for analyses, and the automated scores were calculated as a verification. We observed a positive, strong correlation between the raters' subjective scores and the automated originality scores (i.e., SemiDis; *r*(76) = 0.83, *p* < 0.001).

### 3.3 TF Analysis and CFC During AUT

**FIGURE 4:**
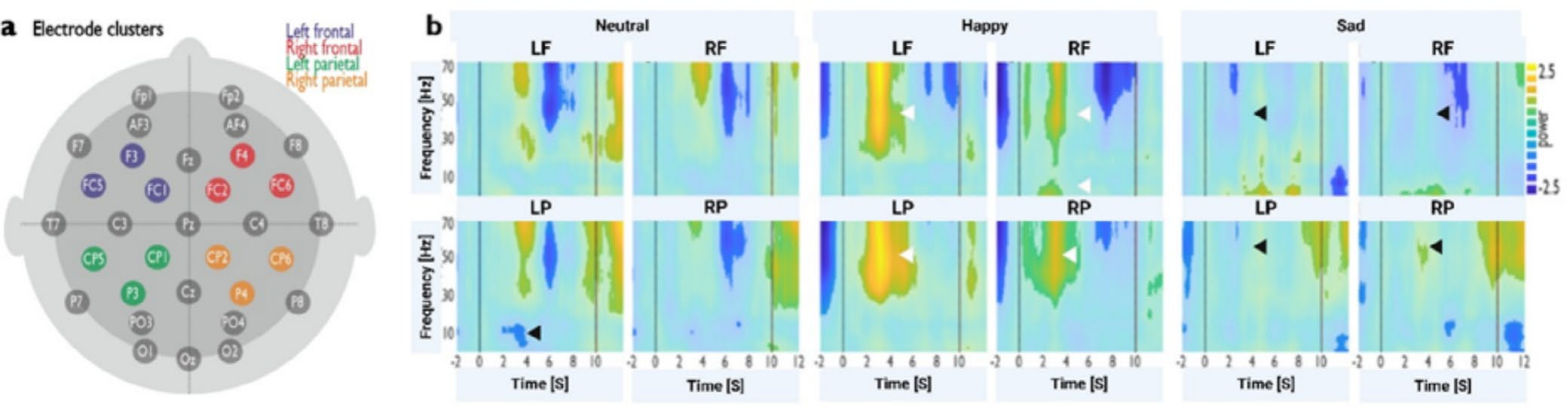
Time‐frequency (TF) plots for EEG data obtained during AUT trials after each emotional induction (neutral, happy, and sad). Panel (a) refers to TF analysis with continuous wavelet transformation, which was performed on single epoched trials for each subject and four different clusters of electrodes over the left frontal (F3, FC5, FC1), right frontal (F4, FC6, FC2), left parietal (P3, CP5, CP1), and right parietal (P4, CP6, CP2) areas. Panel (b) illustrates TF plots for each emotion induction condition represented in the left frontal (LF) and right frontal (RF) areas (upper row) and left parietal (LP) and right parietal (RP) areas (lower row). Each TF plot displays the frequency in Hz (the power fluctuates from 2.5 to −2.5 as signified by the color scale) and the time in seconds on the *y* and *x* axes, respectively. Time 0–10 s indicates the AUT trial window, marked by vertical lines, and the prominent differences between conditions are indicated using arrows.

TF analysis was conducted using continuous wavelet transformation on single‐epoched trials for each subject's reference and activation periods, analyzed across four electrode clusters. These clusters spanned over the left frontal (LF: F3, FC5, FC1), right frontal (RF: F4, FC6, FC2), left parietal (LP: P3, CP5, CP1), and right parietal (RP: P4, CP6, CP2) areas (Figure 4a). Each emotional induction revealed a distinct TF profile during AUT (Figure 4b).

Reduced low‐frequency (delta, theta, and alpha) power was observed in the LP cortex. The happy induction condition was characterized by a stronger gamma power increase relative to baseline in all four cortical regions. However, this gamma power increase was most prominent in the left hemisphere, followed by a less strong reduction in the LP cortex. There was also an increase in low‐frequency power (delta, theta) in the RF cortex 2–4 s after trial onset. Conversely, there was nearly no change in gamma power in the sad induction condition from the reference period to the task period in all four cortical regions. Instead, increased low‐frequency power (theta, delta, and alpha) was observed in the LF cortex.

**FIGURE 5:**
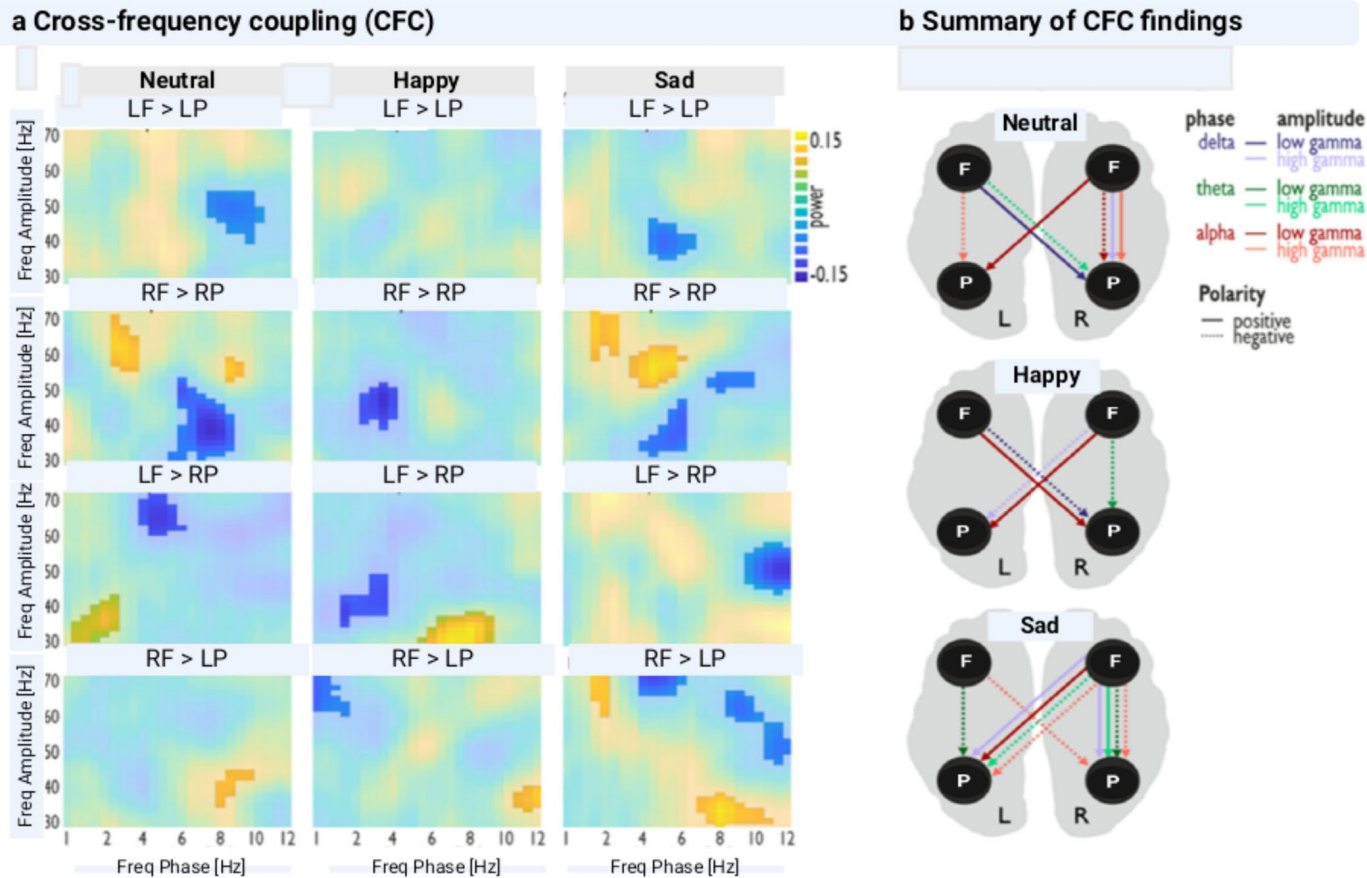
Cross‐frequency coupling (CFC) during AUT after three emotion induction conditions (neutral, happy, and sad). Phase‐amplitude coupling (PAC) paths (rows) are shown for each emotion induction condition (columns) as follows: Left frontal to left parietal (LF–LP), right frontal to the right parietal (RF–RP), left frontal to the right parietal (LF–RP), and right frontal to left parietal (RF–LP) cortex. Each spectrogram displays the frequency of the phase signal in LF and RF on the x‐axes and the frequency of the amplitude signal in LP and RP on the *y*‐axes with its normalized PAC value (range: +0.15 to −0.15; color scale). The PAC value is a normalized measure of how much the phase of the lower‐frequency component modulates the amplitude of the higher‐frequency component. Positive values indicate enhanced modulation, while negative values indicate reduced modulation.

The profiles of PAC during ideational originality for each emotion induction condition are illustrated in Figure 5.

**FIGURE 6:**
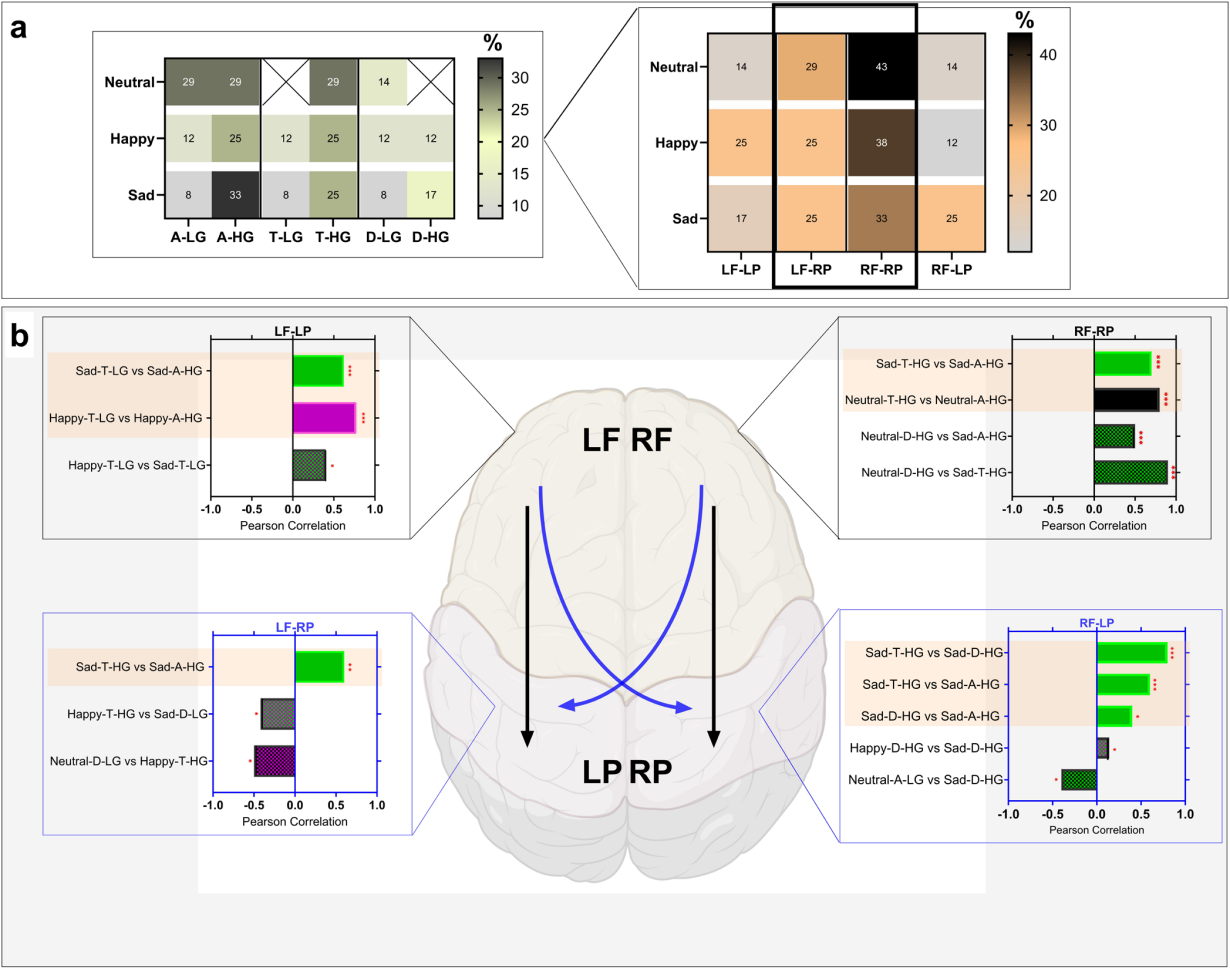
Quantifying frontoparietal PACs distribution during original ideation in three emotion induction conditions (neutral, happy, and sad). Panel (a) refers to the characteristic distribution of the modulation of frontoparietal PACs and its lateralization across the three emotional conditions. Panel (b) refers to the Pearson correlation coefficient (*r*) between the PACs pattern in each frontotemporal network (i.e., LF‐RP, LF‐LP, RP‐LP, and RF‐RP) within and across the three emotional conditions. The highlighted bars signify the correlations within each emotion condition. For a summary, see Table 1. The stars indicate the *p*‐values for the corresponding correlation coefficient (*r*). Stars indicate significance (*p < 0.05, **p < 0.01, ***p < 0.001). Figure S1 illustrates the matrix for Pearson correlations.

A contralateral coupling path with positive polarity was maintained for the three conditions, from RF (alpha) to LP (low gamma), Figure 5. Nevertheless, each condition exhibited a distinctive coupling profile, Figure 5. The identified CFC profiles were analyzed by examining the characteristic correlations within the PAC across three emotional conditions during the original ideation, as shown in Figure 6a. These characterizations reflect the direction and strength of these correlations between frontoparietal PACs, both within the same emotional condition and across them, as presented in Figure 6b and Table 1.

**TABLE 1:** The Pearson correlation coefficient (*r*) between the frontoparietal PACs pattern in each frontotemporal network (i.e., LF‐RP, LF‐LP, RP‐LP, and RF‐RP) within and across the three emotional conditions.

Negative correlation	Coupling			
	LF‐LP	LF‐RP	RF‐RP	RF‐LP
Across emotional induction conditions		Neutral‐D‐LG vs. Happy‐T‐HG		Neutral‐A‐LG vs. Sad‐D‐HG
		Happy‐T‐HG vs. Sad‐D‐LG		

Positive correlation	Coupling			
	LF‐LP	LF‐RP	RF‐RP	RF‐LP
Across emotional induction conditions	Happy‐T‐LG vs. Sad‐T‐LG		Neutral‐D‐HG vs. Sad‐T‐HG	Happy‐D‐HG vs. Sad‐D‐HG
			Neutral‐D‐HG vs. Sad‐A‐HG	
Within emotional condition	Happy‐T‐LG vs. Happy‐A‐HG	Sad‐T‐HG vs. Sad‐A‐HG	Neutral‐T‐HG vs. Neutral‐A‐HG	Sad‐D‐HG vs. Sad‐T‐HG
	Sad‐T‐LG vs. Sad‐A‐HG		Sad‐T‐HG vs. Sad‐A‐HG	Sad‐T‐HG vs. Sad‐A‐HG
				Sad‐D‐HG vs. Sad‐A‐HG

Positive correlations were observed within and across the three emotional induction conditions, Figure 6b, Table 1. Positive correlations were observed within the emotional induction conditions for happy and sad states from the LF to LP cortex. Only positive correlations were found in the sad condition from the LF to RP cortex. Two negative correlations were observed from the LF to RP cortex across emotional conditions: neutral vs. happy and happy vs. sad conditions. Two positive correlations were observed within neutral and sad emotional conditions from the LF to RP cortex, and two positive correlations were similarly observed across these conditions. A positive correlation was observed within the sad emotional condition from the RF to LP cortex.

#### 3.3.1 Neutral Emotion Condition

Relative to the reference period, the neutral condition was characterized by three contralateral couplings and four ipsilateral couplings of both positive and negative polarities, Figure 5a. The two positive contralateral couplings indicated a frontal (alpha and delta) to parietal (low gamma) path. The negative contralateral coupling followed a frontal (theta) to parietal (high gamma) path. The two positive ipsilateral paths were frontal (delta) to parietal (high gamma) and frontal (alpha) to parietal (high gamma). The two negative ipsilateral couplings were frontal (alpha) to parietal (low and high gamma) paths. We observed the following percentages of the frontoparietal PACs lateralization: 14% for LF‐LP, 14% for RF‐LP, 29% for LF‐RP, and 43% for RF‐RP (Figure 6a). Notably, theta modulated by low gamma and delta modulated by high gamma were absent. Instead, the following were observed: alpha modulated by low gamma (29%), alpha modulated by high gamma (29%), theta modulated by high gamma (29%), and delta modulated by high gamma (14%). From RF to RP cortex, a strong positive correlation was found between high‐gamma modulated theta and alpha activity (*r*(24) = 0.84, *p* < 0.001), as depicted in Figure 6b and Table 1.

#### 3.3.2 Happy Emotion Condition

The induced happy condition was characterized by four contralateral couplings with different polarities and one ipsilateral coupling with negative polarity, Figure 5a. The two positive contralateral couplings indicated frontal (alpha) to parietal (low gamma) paths. The two negative contralateral couplings showed frontal (delta) to parietal (low and high gamma) paths. The negative ipsilateral path coupled the frontal (theta) to the parietal (low gamma). We observed the following percentages of the frontoparietal PACs lateralization: 25% for LF‐LP, 12% for RF‐LP, 25% for LF‐RP, and 38% for RF‐RP (Figure 6a). In contrast to the neutral conditions, theta modulated by low gamma and delta modulated by high gamma were expressed at 12% each. The percentages for modulations were alpha modulated by low gamma (12%), alpha modulated by high gamma (25%), theta modulated by high gamma (25%), and delta modulated by high gamma (12%). A strong positive correlation was found between low‐gamma modulated theta activity and high‐gamma modulated alpha activity from the LF to LP cortex (*r*(24) = 0.77, *p* < 0.001), as depicted in Figure 6b and Table 1.

#### 3.3.3 Sad Emotion Condition

The sad condition was characterized by five contralateral and five ipsilateral couplings with different polarities, Figure 5a. The two positive contralateral couplings revealed frontal (alpha and delta) to parietal (low and high gamma) paths. The three negative contralateral couplings indicated frontal (alpha and theta) to parietal (high gamma) paths. The two positive ipsilateral couplings showed frontal (delta and theta) to parietal (high gamma) paths. The three negative ipsilateral paths were frontal (theta and alpha) to parietal (low and high gamma). We observed the following percentages of the frontoparietal PACs lateralization: 17% for LF‐LP, 25% for RF‐LP, 25% for LF‐RP, and 33% for RF‐RP (Figure 6a). Unlike the neutral conditions, theta modulated by low gamma and delta modulated by high gamma were observed at 8% and 17%, respectively. The percentages for the various modulations were alpha modulated by low gamma (8%), alpha modulated by high gamma (33%), theta modulated by high gamma (25%), and delta modulated by high gamma (8%). There was a moderate positive correlation between theta modulated by low gamma and alpha modulated by high gamma from the LF to LP cortex (*r*(24) = 0.62, *p* = 0.001), as depicted in Figure 6b and Table 1. Moderate positive correlations were found from the LF to RP cortex between high gamma‐modulated alpha and theta oscillations (*r*(24) = 0.63, *p* = 0.001), as depicted in Figure 6b and Table 1. A strong positive correlation was observed between theta and alpha activity modulated by high gamma from the RF to RP cortex (*r*(24) = 0.71, *p* < 0.001), as depicted in Figure 6b and Table 1. The following differential positive correlations were identified from the RF to LP cortex: two moderate positive correlations between high gamma‐modulated theta and alpha oscillations (*r*(24) = 0.58, *p* = 0.002), as depicted in Figure 6b and Table 1, and between high gamma‐modulated delta and alpha activity (*r*(24) = 0.43, *p* = 0.03), as depicted in Figure 6b and Table 1. A strong positive correlation was found between high gamma‐modulated theta and delta activity (*r*(24) = 0.79, *p* < 0.001), as depicted in Figure 6b and Table 1.

#### 3.3.4 Across Emotion Conditions

A moderate positive correlation between the low gamma modulation of theta was observed in the happy and sad emotion conditions from the LF to LP cortex (*r*(24) = 0.41, *p* = 0.04), as depicted in Figure 6b and Table 1. Two moderate negative correlations were observed across induced emotion conditions from the LF to RP cortex: neutral vs. happy states and happy vs. sad states. These moderate negative correlations were as follows: 1‐between low gamma‐modulated delta activity in the neutral condition and high gamma‐modulated theta in the happy condition (*r*(24) = −0.47, *p* = 0.01), as depicted in Figure 6b and Table 1, and 2‐between high gamma‐modulated theta in the happy condition and low gamma‐modulated delta in the sad condition (*r*(24) = −0.42, *p* = 0.03), as depicted in Figure 6b and Table 1. From the RF to RP cortex, two differential positive correlations were observed. A moderate positive correlation was between high gamma‐modulated delta in the neutral and high gamma‐modulated alpha in the sad condition (*r*(24) = 0.54, *p* < 0.001), as depicted in Figure 6b and Table 1. A strong positive correlation was between high gamma‐modulated delta in the neutral and high gamma‐modulated theta in the sad condition (*r*(24) = 0.91, *p* < 0.001), as depicted in Figure 6b and Table 1. From the RF to LP cortex, a negative correlation was found between the neutral and sad emotional conditions and a strong positive correlation between the happy and sad conditions. This negative correlation was moderately present between the low gamma‐modulated alpha of the neutral and high gamma‐modulated delta of the sad condition (*r*(24) = −0.42, *p* = 0.03), as depicted in Figure 6b and Table 1. A weak positive correlation was observed between high gamma‐modulated delta in the happy and sad conditions (*r*(24) = 0.38, *p* = 0.05), as depicted in Figure 6b and Table 1.

### 3.4 Exploratory Analysis: Ideational Originality, Neural Dynamics, and Gender

**FIGURE 7:**
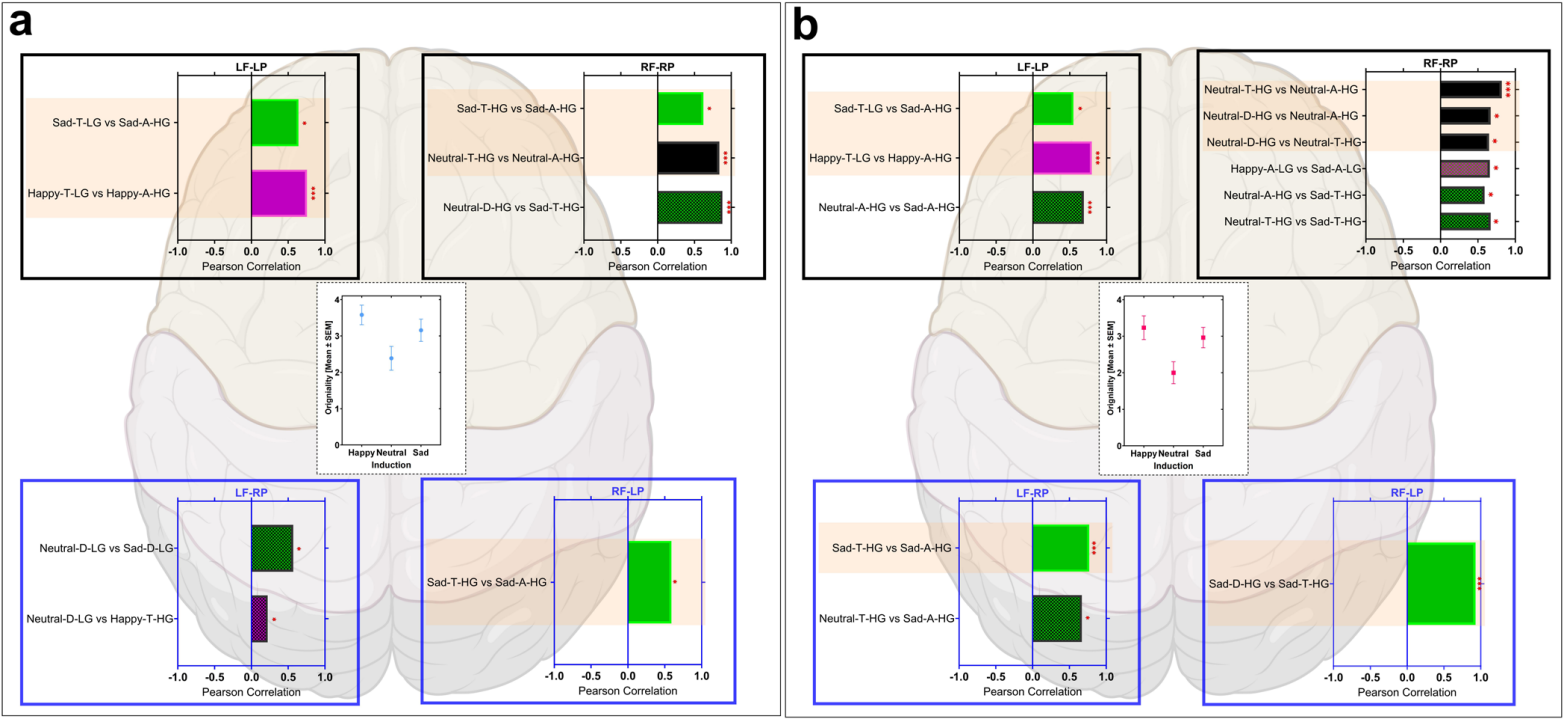
Gender Pearson correlation coefficient between frontoparietal PACs pattern in each frontoparietal network (i.e., LF‐RP, LF‐LP, RP‐LP, and RF‐RP) across the three emotional conditions. Pearson correlation coefficient between the CFC pattern in each coupling (i.e., LF‐RP, LF‐LP, RP‐LP, and RF‐RP) across the three emotional conditions for males (panel (a)) and females (panel (b)). The highlighted bars signify the correlations within each emotion condition. For a summary, see Table 2. The stars indicate the *p*‐values for the corresponding correlation coefficient. Stars indicate significance (*p < 0.05, **p < 0.01, ***p < 0.001). Figures S2 and S3 illustrate the matrices for Pearson correlations.

The exploratory gender profiles of the neural dynamics indicate the strength of correlations between PAC values along each path for each induced emotion condition during ideational originality, as shown in Figure 7 and Table 2.

**TABLE 2:** The Gender Pearson correlation coefficient (*r*) between the frontoparietal PACs pattern in each frontotemporal network (i.e., LF‐RP, LF‐LP, RP‐LP, and RF‐RP) within and across the three emotional conditions.

Gender	Positive correlation	Coupling			
		LF‐LP	LF‐RP	RF‐RP	RF‐LP
Male	Across emotional induction conditions		Neutral‐D‐LG vs. Happy‐T‐HG	Neutral‐D‐HG vs. Sad‐T‐HG	
			Neutral‐D‐LG vs. Sad‐D‐LG		
	Within emotional condition	Happy‐T‐LG vs. Happy‐A‐HG		Neutral‐T‐HG vs. Neutral‐A‐HG	Sad‐T‐HG vs. Sad‐A‐HG
		Sad‐T‐LG vs. Sad‐A‐HG		Sad‐T‐HG vs. Sad‐A‐HG	
Female	Across emotional induction conditions	Neutral‐A‐HG vs. Sad‐A‐HG	Neutral‐T‐HG vs. Sad‐A‐HG	Neutral‐T‐HG vs. Sad‐T‐HG	
			Sad‐A‐HG vs. Sad‐T‐HG	Neutral‐A‐HG vs. Sad‐T‐HG	
	Within emotional condition			Happy‐A‐LG vs. Sad‐A‐LG	
		Happy‐A‐HG vs. Happy‐T‐LG		Neutral‐D‐HG vs. Neutral‐T‐HG	
		Sad‐T‐LG vs. Sad‐A‐HG		Neutral‐D‐HG vs. Neutral‐A‐HG	
				Neutral‐T‐HG vs. Neutral‐A‐HG	

A similar trend was observed on the behavioral level: originality was highest in the happy condition, lower in the sad condition, and lowest in the neutral condition. However, there were no significant differences, as illustrated in Figure 7. This might be due to the low sample size (i.e., 13 males vs. 13 females). The neural dynamics of male and female participants exhibited different correlation profiles across the four lateralization paths (LF to LP, LF to RP, RF to RP, and RF to LP). These profiles are presented in Figure 7 and Table 2. Notably, there were more correlations along the path from RF to RP in males and females than along other lateralization paths. There were fewer correlations along the path from RF to LP. Moreover, females expressed a higher number of correlations in the lateralization from RF to RP than males, and all of these correlations were significantly positive.

The correlation profiles for the RF to LP path in sad condition were similar across genders. Males demonstrated a strong correlation between low gamma‐modulated theta and high gamma‐modulated alpha (*r*(11) = 0.66, *p* = 0.01), as shown in Figure 7a and Table 2. Females exhibited a moderate correlation between low gamma‐modulated theta and high gamma‐modulated alpha (*r*(11) = 0.55, *p* = 0.05), illustrated in Figure 7b and Table 2. In the happy condition, males displayed an even stronger correlation between low gamma‐modulated theta and high gamma‐modulated alpha (*r*(11) = 0.74, *p* < 0.0001), as seen in Figure 7a and Table 2. Females also showed a strong positive correlation in the happy condition between low gamma‐modulated theta and high gamma‐modulated alpha (*r*(11) = 0.80, *p* < 0.0001), detailed in Figure 7b and Table 2. Female participants exhibited a strong positive correlation between high gamma‐modulated alpha in the neutral and sad conditions (*r*(11) = 0.69, *p* = 0.01), as depicted in Figure 7b and Table 2.

A positive correlation was observed in the coupling from LF to RP cortices in both males and females, as shown in Figure 7 and Table 2. This correlation was found in males across different emotional conditions, as illustrated in Figure 7a and Table 2. In contrast, females exhibited this correlation primarily within and across the sad condition, as illustrated in Figure 7b and Table 2. In male participants, the LF to RP path indicated a moderate negative correlation between low gamma‐modulated delta in the neutral condition and high gamma‐modulated theta in the happy condition (*r*(11) = −0.57, *p* = 0.04), as described in Figure 7a and Table 2. There was a moderate positive correlation between low gamma‐modulated delta in the neutral and sad conditions (*r*(11) = 0.57, *p* = 0.04), as depicted in Figure 7a and Table 2. For female participants, strong positive correlations were found between high gamma‐modulated theta in the neutral condition and high gamma‐modulated alpha activity in the sad condition (*r*(11) = 0.67, *p* = 0.01), as shown in Figure 7b and Table 2. Furthermore, a strong positive correlation was observed between high gamma‐modulated theta and alpha in the sad condition (*r*(11) = 0.77, *p* < 0.0001), described in Figure 7b and Table 2.

In the male participants, a positive correlation was observed in the path from RF to RP, both within the neutral and sad conditions (Figure 7a). Specifically, strong and moderate positive correlations were found between high gamma‐modulated alpha and theta oscillations in both the neutral (*r*(11) = 0.84, *p* < 0.001) and sad (*r*(11) = 0.62, *p* = 0.02) conditions, respectively, as shown in Figure 7a and Table 2. A strong positive correlation was observed between the high gamma‐modulated delta activity in the neutral condition and the high gamma‐modulated theta activity in the sad condition (*r*(11) = 0.88, *p* < 0.001). In female participants, three positive correlations were identified within the neutral condition (depicted in Figure 7b and Table 2). These correlations included a moderate correlation between high gamma‐modulated delta and theta oscillations (*r*(11) = 0.65, *p* = 0.02), a strong correlation between high gamma‐modulated alpha and theta (*r*(11) = 0.82, *p* = 0.001), and a moderate correlation between high gamma‐modulated alpha and delta (*r*(11) = 0.67, *p* = 0.01). Furthermore, three positive correlations were observed across the neutral and sad conditions (as shown in Figure 7b and Table 2). A moderate positive correlation was observed between high gamma‐modulated alpha in the neutral condition and high gamma‐modulated theta in the sad condition (*r*(11) = 0.59, *p* = 0.03). Strong positive correlations were found between high gamma‐modulated theta in the neutral condition and high gamma‐modulated theta in the sad condition (*r*(11) = 0.67, *p* = 0.01), as well as between high gamma‐modulated delta in the neutral condition and high gamma‐modulated theta in the sad condition (*r*(11) = 0.92, *p* < 0.001). Lastly, there was a strong positive correlation between low gamma‐modulated alpha in the happy and sad conditions (*r*(11) = 0.66, *p* = 0.01). In males, a moderate positive correlation was found in the RF to LP path between high gamma‐modulated theta and alpha oscillations during the sad condition (*r*(11) = 0.59, *p* = 0.03), as shown in Figure 7a and Table 2. In females, a strong positive correlation was identified between high gamma‐modulated theta and delta in the sad condition (*r*(11) = 0.93, *p* < 0.001), illustrated in Figure 7b and Table 2.

## 4 Discussion

**FIGURE 8:**
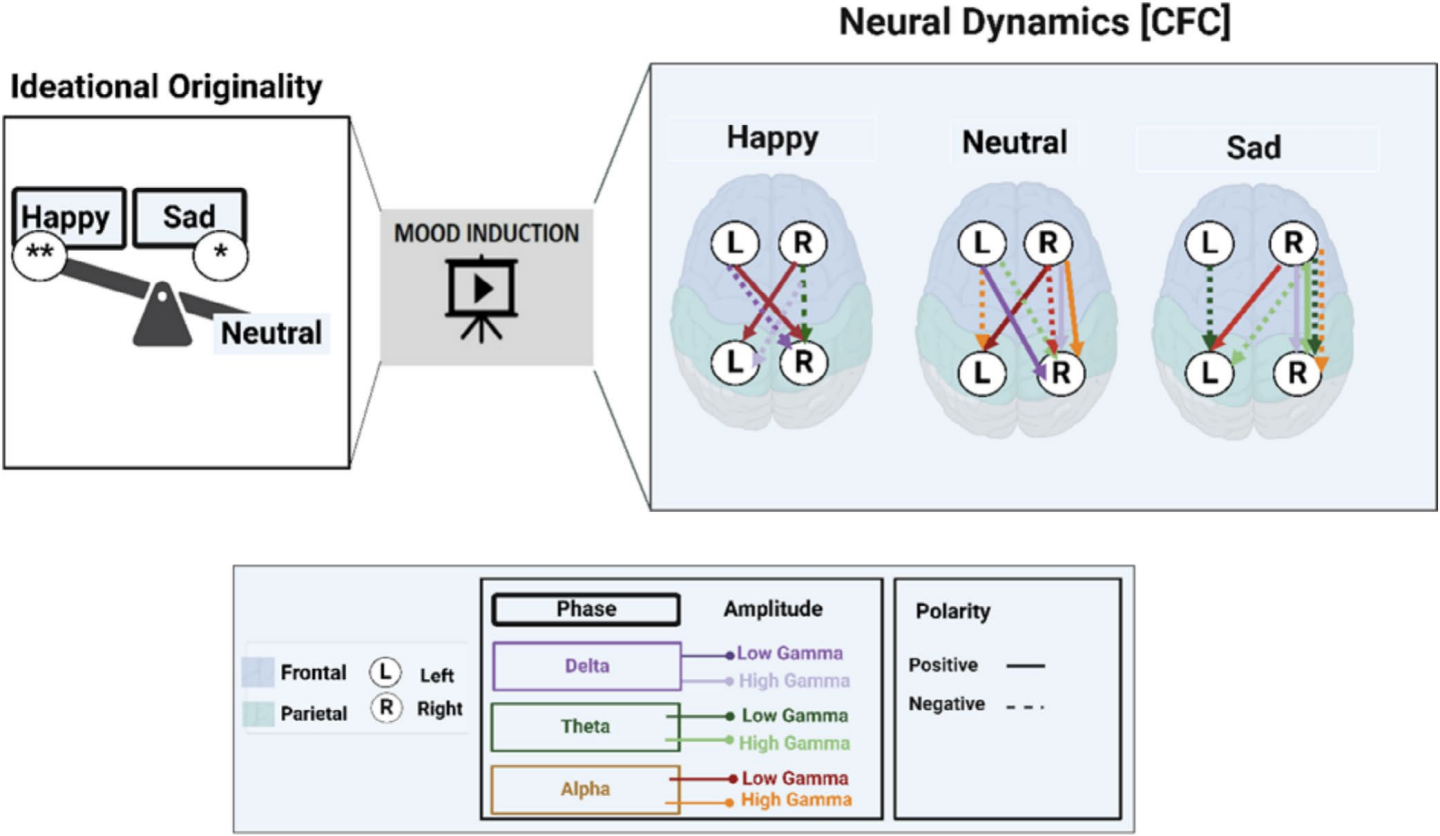
Summary of the effects of emotion induction on original ideation and associated neural dynamics. The behavioral level represents the original ideation scores that express a significant difference between happy and neutral states and between sad and neutral states. The neural dynamics of cross‐frequency coupling (CFC) are illustrated as a function of phase‐amplitude coupling (PAC) across happy, neutral, and sad states during ideational originality. The legend describes coupling indicators, including phase, amplitude, and polarity.

Our findings shed light on the influence of emotional inductions (neutral, sad, and happy) on originality and associated neural dynamics during ideational originality. We have summarized the effects of these emotional inductions on original ideation and characterized the related functional connectivity (i.e., TF and PAC profiles) in Figure 8.

At the behavioral level, subjective ratings of arousal and pleasure confirmed the effective induction of emotional states and their influence on ideational originality. These findings support the *activating hypothesis* (Amabile 1983; de Dreu et al. 2008; Simonton 1997). Ideational originality significantly increased in happy and sad emotion induction conditions compared to neutral induction. Increasing ideational originality after happy induction may suggest that individuals experience more fun or joy (Hirt et al. 1997). Previous research reported an association between positive mood and DA, which facilitates creative thinking (Baas et al. 2008; Davis 2009) and ideational originality (Agnoli et al. 2023; Chermahini and Hommel 2010, 2012; de Rooij and Vromans 2020; Ueda et al. 2016).

The reconciliation of sadness with enhanced originality suggests that it is not just the valence of an emotional state that influences original ideation but also the level of arousal and the cognitive pathway it promotes (de Dreu et al. 2008; He et al. 2017; Nijstad et al. 2010; Xiao et al. 2023). When interpreting our behavioral findings, we must emphasize that we specifically focused on the original idea generation of divergent creative thinking in young adults. Our findings represent a younger demographic than individuals with advanced professional creative skills, which differ from those in our study (Agnoli et al. 2018). Moreover, our results may not align with figural measures of creativity, as emphasized by the recent meta‐analysis of Grajzel et al. (2023).

### 4.1 Neural Dynamics

Emotional induction elicited distinct neural fronto‐parietal coupling profiles during original ideation. These signature patterns depict how inducing positive, neutral, and negative emotional inductions influences information processing when generating original ideas.

#### 4.1.1 TF Profiles

The neutral condition was characterized by increased gamma between 2 and 4 s after trial onset relative to the reference period, particularly in the left hemisphere. This increase was followed by a decrease in gamma activity in both hemispheres. The happy condition was characterized by a considerably stronger gamma increase in the early time window, 2–4 s after trial onset. This enhanced gamma activity might suggest the execution of WM (Yamamoto et al. 2014). Its transient nature and subsequent decrease could indicate a prompt engagement in solving insight problems (Arden et al. 2010).

The prevalence of gamma activity in the left and right frontal and parietal cortices (LF, RF, LP, and RP), with a more substantial presence in the left hemisphere, suggests a bias towards the left hemisphere in WM loading (Roux and Uhlhaas 2014) during original ideation (Benedek et al. 2023). The increase in low‐frequency oscillations (theta and delta) observed in RF may indicate the demand for RF for top‐down control (Canolty et al. 2006). The increase in low‐frequency oscillations (theta, delta, and alpha in LF) may arise from heightened inhibitory activity that disrupts ongoing gamma activity to regulate sensory inputs (Jensen and Mazaheri 2010; Payne and Kounios 2009; Worden et al. 2000).

#### 4.1.2 PAC Profiles

Functional connectivity couplings of happy induction conditions suggest suppressing well‐established associations while strengthening weaker ones (Vaz et al. 2017). The reduced number of PAC paths (i.e., fronto‐parietal couplings) during the happy condition could indicate access to a broader and more diverse range of information, activating more material from WM (Isen 1993). The modulation of gamma activity by the low‐frequency delta and theta waves was observed across different fronto‐parietal networks in happy and sad conditions at higher percentages than in neutral conditions. These modulations indicate efficient information transmission across fronto‐parietal networks during two activated emotional states while generating original ideas to assist the integration of sensory information with EC (Senkowski and Engel 2024).

The percentages of frontoparietal PACs' lateralization and modulations differed across the three emotional conditions; however, the variations were similar in happy and sad conditions but different from the neutral condition. These variations suggest that inducing happiness and sadness can enhance WM through different processing modes—spontaneous and deliberate—for generating original ideas (Dietrich 2004). The occurrence of delta modulated by high‐gamma PAC only in happy and sad emotional conditions, Figure 5, suggests that the attention system is involved in storing information in these two conditions (Roux and Uhlhaas 2014).

All three emotional conditions preserved a common coupling path: the coupling of low gamma activity in LP to the phase of the contralateral RF alpha. The maintenance of these sharp waveforms^3^ in all three emotional conditions supports the notion that alpha‐gamma PAC is necessary for WM function (Roux and Uhlhaas 2014), gating sensory information (Jensen and Mazaheri 2010), imagined actions (de Lange et al. 2008), and blocking out distraction (Bonnefond and Jensen 2013) during ideational originality. The association of increased frontoparietal PACs between frontal delta, theta, and parietal gamma suggests WM maintenance (Tóth et al. 2012; Tseng et al. 2019) and also implies attentional control and the suppression of irrelevant information (Bonnefond and Jensen 2013; Jensen and Mazaheri 2010; O'Rourke et al. 2015). The variations observed in coupling paths within fronto‐parietal networks suggest that the process of original ideation is likely contingent upon WM, the retrieval and maintenance of memory, and the involvement of further control mechanisms (Stevens and Zabelina 2019).

The positive correlations between frontoparietal PACs within three emotion conditions suggest enhanced interregional coupling during ideational originality. Conversely, the negative correlations between frontoparietal PACs across these conditions may indicate potential competition or inhibition (Roux and Uhlhaas 2014; Solomon et al. 2017) among various emotional conditions during original ideation. The positive correlations across these three conditions indicate that the brain maintains specific functional connectivity couplings that support cognitive processing of original ideation, regardless of the emotional context. The positive correlation between high gamma‐modulated theta and alpha oscillations in the neutral condition—especially from LF to RP cortex—reflects the role of high‐frequency gamma activity in monitoring WM storage in distant sensory areas of the parietal cortex (Daume et al. 2017; Lara and Wallis 2014) during original ideation.

The positive correlation between theta and alpha activity—modulated by low and high gamma, respectively—observed from the LF to LP cortex during the happy condition suggests that coordinated interaction between activity in different frequency bands may facilitate cognitive processes such as WM and attention, ultimately leading to exploring the generation of original ideas through cognitive flexibility (Nijstad et al. 2010). This neural coupling could explain that happy emotion induction may lead to automatic processing during ideational originality, which allows exploring original ideas to be loaded in WM and silencing the attentional system (Dietrich 2004). The silence of the attentional system could allow for storing information in WM, which becomes more active after inducing sad emotion to maintain items in WM (Isen 1993). Accordingly, the distinctive frontopartial PAC patterns observed in the case of the sad induction condition might be interpreted as the sad condition being comparatively biased toward the deliberate (control) processing mode instead of the automatic one during original ideation (Dietrich 2004).

Positive correlations between theta, alpha, and delta oscillations modulated by gamma frequencies in the sad induction condition indicate a dynamic interplay of neural mechanisms that support the exploitation of original idea generation (Charnov 1976; Hills, Todd, Jones, et al. 2015; Hills and Kenett 2025; Hills and Pachur 2012; Todd and Hills 2020). Whether exploratory or exploitative, search processes in creative cognition (Fauconnier and Turner 1998; Simonton 1997; Ward et al. 1997) play a vital role in generating novel ideas (Hills et al. 2008). Thus, negative emotion may catalyze creative ideation through cognitive persistence (de Dreu et al. 2008; Nijstad et al. 2010). In the same vein, positive correlations from the LF cortex to the RP cortex following the induction of sad emotion suggest that sadness activates extensive frontoparietal networks for emotional processing, which may enhance cross‐hemispheric communication and increase the demand for cognitive resources, leading to guiding selectivity of original ideas during the exploitation phase (Charnov 1976; Hills, Todd, Jones, et al. 2015; Hills and Kenett 2025; Hills and Pachur 2012; Todd and Hills 2020).

The positive correlations between the LF and LP cortex after the induction of happy and sad emotions indicate that both emotional inductions promote similar, although distinct, patterns of neural activation associated with original ideation. Different paths for generating these original ideas involve overlapping neural networks from frontal to parietal regions through exploration and exploitation (Charnov 1976; Hills, Todd, Jones, et al. 2015; Hills and Kenett 2025; Hills and Pachur 2012; Todd and Hills 2020). Therefore, when individuals experience happiness or sadness, a coordinated neural response may facilitate emotional processing and cognitive engagement differently through these two pathways of exploration and exploitation for generating original ideas. These two pathways echo Nijstad's dual paths of creativity (Nijstad et al. 2010), that is, cognitive flexibility and persistence. Interestingly, the positive correlation between low gamma‐modulated theta in happy and sad conditions from the LF to LP regions suggests that a shared neural mechanism engages these oscillations in both contexts despite the contrasting emotional valences.

The negative correlations from LF to RP cortex across emotional induction conditions (i.e., between neutral vs. happy and between happy vs. sad) imply that cognitive processing associated with these emotional conditions may compete for cognitive resources or be inversely related to original idea generation. Consequently, the dynamics of cognitive processing of generating original ideas are influenced by emotional context, which contributes to contrasting neural responses that facilitate original idea generation. The two negative correlations between neutral vs. happy and happy vs. sad induction—from LF to RP cortex—may indicate a cognitive shift wherein engagement in one emotional condition (e.g., happy) detracts from the processing of another (e.g., neutral or sad), which is explained through cognitive flexibility and persistence (i.e., the dual pathways to creativity model; Nijstad et al. 2010). The enhancement of original idea generation during the happy condition could be explained through cognitive flexibility (Nijstad et al. 2010), which is associated with exploring a wide range of original ideas (Hills et al. 2008; Hills, Todd, Lazer, et al. 2015; Hills and Kenett 2025; Todd and Hills 2020). In contrast, cognitive persistence (Nijstad et al. 2010) may explain the increased generation of original ideas during the sad condition compared to the neutral condition through focused and exploitative processing (Hills et al. 2008; Hills, Todd, Lazer, et al. 2015; Hills and Kenett 2025; Todd and Hills 2020); this might explain the observation of fewer original ideas compared to the happy induction condition.

In conclusion, our findings suggest the brain leverages emotional depth to enhance cognitive flexibility (happy induction condition) and cognitive persistence (sad induction condition), allowing it to generate novel ideas through exploration and exploitation paths, respectively. The dual‐pathway to creativity model (Nijstad et al. 2010) can explain the reconciliation of sadness with enhanced originality during the exploitative phase through the cognitive persistence pathway. Thus, although sadness is typically viewed as a deactivating emotion that inhibits originality, it may enhance originality when induced to elevate cognitive persistence (Nijstad et al. 2010) and controlled processing (de Dreu et al. 2008).

The negative correlations identified between neutral delta and happy theta and between happy theta and sad delta suggest that the brain's response to different emotional inductions might inhibit certain oscillatory activities (Jensen and Mazaheri 2010; Payne and Kounios 2009; Worden et al. 2000). The negative correlation between neutral‐low gamma‐modulated alpha and sad‐high gamma‐modulated delta from RF to LP cortex indicates that as individuals experience sadness, there is a decrease in alpha activity associated with attentional control and inhibition of irrelevant information (Bonnefond and Jensen 2013; Jensen and Mazaheri 2010; O'Rourke et al. 2015). Conversely, the positive correlation between high gamma‐modulated delta in the happy and sad conditions indicates that the two emotional states may share underlying neural mechanisms engaging delta oscillations, indicating common emotional processing pathways supporting ideational originality, which involves WM (Daume et al. 2017; Lara and Wallis 2014; Murphy et al. 2020; Palva et al. 2010; Roux and Uhlhaas 2014; Yamamoto et al. 2014) and attention (Beaty et al. 2016, 2018, 2019; Gonen‐Yaacovi et al. 2013; Rominger et al. 2019).

However, we could not rule out the common challenges related to interpreting neural coupling of cognitive functions, which arise from the multifunctionality of brain networks and dynamic neural reconfigurations. Brain networks are not exclusively dedicated to single cognitive tasks (Menon 2011), that is, the DN network, often linked to mind‐wandering or self‐referential thoughts, also contributes to episodic memory and creative thinking. Neural coupling patterns are not static but change depending on task complexity, attention, and emotional states; thus, the brain dynamically reconfigures itself based on task demands (Bassett and Sporns 2017). This dynamic nature of neural coupling patterns implies that a given coupling pattern, such as increased connectivity between prefrontal and parietal areas during WM tasks, may not exclusively reflect WM but also encompass attentional control, motivation, or emotional processing. Numerous cognitive processes, including WM and word association, overlap brain regions and neural patterns (Miller and Cohen 2001). It is challenging to discern whether this activation is specific to a particular cognitive process or is the result of multiple intertwined processes. Therefore, asserting that a specific neural coupling pattern exclusively corresponds to a single cognitive function, such as WM or word association, is challenging. Accordingly, it is imperative to conduct follow‐up studies to validate these relationships.

To the best of our knowledge, this study is the first to report the influence of three basic emotional inductions on the ideation process as a function of originality and associated neural dynamics. Our findings contribute to establishing a foundational framework that advances neurocognitive and behavioral research on emotional induction and ideational originality. We anticipate that future subsequent studies will leverage this framework to address the limitations identified in the current study.

#### 4.1.3 Exploratory Overview: Ideational Originality, Neural Dynamics, and Gender

Our exploratory correlation analysis of frontoparietal PACs during ideational originality across various emotional conditions emphasizes significant gender differences. Our exploratory overview^4^ provides directions for future research on gender differences in ideational originality. These results suggest a potential explanation for how neural functional connectivity related to emotional and cognitive processing of ideational originality varies between genders.

The correlation analysis of frontoparietal PACs—LF to LP, LF to RP, RF to RP, and RF to LP paths—suggests that males and females operate distinct neural coupling paths when engaging in original ideation, particularly in response to different emotional conditions. Although no study focuses on neural dynamics and original ideation in the context of emotion induction, earlier studies (Abraham 2016; Abraham et al. 2014; He and Wong 2021; Ivcevic et al. 2022; Silberstein et al. 2019) indicated cognitive styles and neural mechanisms that might be operated differently across gender during creative thinking.

The higher number of significant correlations from the RF to RP in both genders than in other lateralization paths indicates that this specific neural path of the right hemisphere is crucial for original ideation processing. Moreover, there are more correlations in females in this right hemispheric path than in males, suggesting that women may engage more bias with the neural mechanisms associated with right hemispheric lateralization of the frontotemporal network during ideational originality. The positive correlations between low gamma‐modulated theta and high gamma‐modulated alpha across emotional conditions for both genders suggest shared neural mechanisms that support ideational originality. Theta oscillations are associated with cognitive control and emotional processing, while alpha oscillations are associated with attentional focus and inhibition of distractions (Roux and Uhlhaas 2014; Solomon et al. 2017).

Interestingly The negative correlation observed in males between neutral‐low gamma‐modulated delta and happy‐high gamma‐modulated theta may indicate a shift in cognitive resources towards more active emotional processing. This negative correlation suggests that males experience a more pronounced modulation of their neural activity in response to emotional changes. Conversely, females exhibit a positive correlation between neutral‐high gamma‐modulated theta and sad‐high gamma‐modulated alpha, implying that females maintain a more steady neural response across different emotional induction conditions.

The path from RF to LP showed positive correlations for both genders, suggesting the functional role of this path in processing emotional information across various conditions. In males, the positive correlation between high gamma‐modulated theta and alpha during the sad condition indicates that they may use this path to manage their emotional processing during ideational originality. In contrast, the positive correlation in females between high gamma‐modulated theta and delta during the sad condition reveals a different neural strategy, where females may engage both high‐ and low‐frequency oscillations to guide their emotional experiences.

While both genders exhibit positive correlations in certain neural coupling paths, correlation strength and pattern variations suggest that gender may affect how emotional experiences are processed and integrated within the neural frontoparietal networks involved in ideational originality. Thus, considering gender differences in brain‐behavioral research is useful, particularly in studies examining the interaction of emotional and cognitive processes during creative ideation.

## 5 Limitations, Open Questions, and Future Directions

Over the past decade, an increasing interest has been in understanding how emotional states affect creative thinking (Baas 2015; Baas et al. 2008; de Dreu et al. 2008). However, the brain's neural dynamics of this research question are still open to further investigation. As such, our findings should be interpreted and discussed with an awareness of their limitations.

Our relatively small sample size of university students should be replicated in the future with a larger sample size, particularly to examine the effect of gender. Due to the within‐subject design, there might still be effects on the order of the videos. I.e., ruminations about the sad and happy conditions are possible during the neutral condition. Although the SAM scale after each video to assess arousal and pleasure levels indicates the effectiveness of the emotional induction, we cannot dismiss the possibility that participants may have felt bored or experienced mixed emotions. We focused only on happy and sad emotion induction but could expand to include joy, anger, and contentment to provide a broader framework for the relationship between emotion induction with different intensities, ideational originality, and underlying neural dynamics. Previous research has shown that optimism intensifies positive emotions (i.e., enthusiasm and happiness) and diminishes negative emotions (i.e., sadness and fear) (Baas 2015; Baas et al. 2008).

The imposed time constraints during EEG in our experiment may have limited the behavioral dimensions scoring of creative ideation and did not allow for measuring fluency and flexibility. The reason is that the task was instructed to generate original ideas (i.e., original uses) rather than alternative uses, which could be counted for fluency and flexibility. Furthermore, ideational originality requires time to emerge, so only low levels of originality can be captured in this short experiment duration (i.e., 10 s). Therefore, future replication of this experiment using longer response durations will allow participants to provide more original responses.

We statistically analyzed EEG data only at the group level, and no individual data that could be associated with individual behavioral data were extracted. Addressing this issue in the context of individual differences would provide valuable insights for future research. Extending this study by examining the effect of sEBR changes as an indicator of phasic DA changes on ideational originality in association with the underlying dynamical neural mechanisms is another noteworthy direction to consider (Agnoli et al. 2023; Chermahini and Hommel 2010, 2012; de Rooij and Vromans 2020; Ueda et al. 2016). Brain neural networks are multifunctional (Murphy et al. 2020), so we must exercise caution when interpreting them to avoid overgeneralization.

In this regard, valuing hypotheses regarding neural coupling patterns and their correlation with original ideation could be beneficial. We could include both EC‐related task(s) and a battery of DT tasks to strengthen the interpretation of our current findings. It is also beneficial to consider intelligence components, as previous research has indicated strong associations between intelligence components and DT (Benedek et al. 2014; Benedek, Franz, et al. 2012; Benedek, Könen, et al. 2012; Nusbaum and Silvia 2011; Silvia et al. 2013). Finally, we should consider that various DT tasks assess slightly different dimensions of DT, that is, when comparing verbal and figural measures (Abraham and Bubic 2015; Byrne 2002; Clapham 2004; Hass and Beaty 2018; Silvia 2011).

These limitations were unavoidable because no prior study had used functional connectivity coupling to study the influence of emotion induction on ideational originality and the associated neural dynamics. Accordingly, it is imperative to conduct follow‐up studies to determine the specificity of the activation of functional coupling patterns to a particular cognitive process of original ideation or the result of multiple intertwined processes in the context of emotion induction.

## Author Contributions

R.K., S.F., and B.G. designed the research and the experimental design. R.K. conducted the experiment. R.K., B.G., and S.F. performed the analysis. R.K. drafted the manuscript with significant insights from S.F. and B.G.

## Conflicts of Interest

The authors declare no conflicts of interest.

## Supporting information


**Figure S1.** Pearson correlation matrix for Figure 6.

**Figure S2.** Pearson correlation matrix for Figure 7a.

**Figure S3.** Pearson correlation matrix for Figure 7b.

## Data Availability

The data that support the findings of this study are available from the corresponding author upon reasonable request.

## Endnotes

^1^
EEG has been widely used to study brain activity by measuring electrical signals produced by neural oscillations, and it allows a more fine‐grained temporal analysis of brain activation in response to specific cognitive events, albeit with lower spatial resolution than fMRI (Fink and Benedek 2014; Srinivasan 2007). These neural oscillations occur at various frequencies and are critical for cognitive and functional processes.

^2^
Functional connectivity analysis might serve as a neural gating mechanism, where the phase of low‐frequency oscillations regulates the occurrence of high‐frequency bursts of activity (Senkowski and Engel 2024). This gating mechanism enables the brain to prioritize certain neural processes or inputs over others, particularly in tasks requiring high‐order executive functions.

^3^
Traditionally, PACs between the alpha phase and low gamma amplitude are called “sharp waveforms” (Vaz et al. 2017).

^4^
These exploratory results should be read cautiously due to the relatively low sample size between gender.

## Experimental Stimuli of AUT

**Table:**

Spoon	Newspaper	Sock
Knife	Comb	Lighter
Cup	Lipstick	Hat
Pen	Candle	Bottle
Toothbrush	Bucket	Hanger
Shoe	Clock	Tire
Brick	Belt	Barrel
Paper‐clip	Cork	Hammer
Broom	Fork	Book
Lamp	Wine opener	Teapot
Spong	Basket	Trash pin
Screwdriver	Shop stick	Fan
Mirror	Tin	Umbrella
Scarf	Dental floss	Toilet paper
Soap	Button	Scissor
