# Supplementary figures and images for: Emotion Induction Modulates Neural Dynamics Related to the Originality of Ideational Creativity
Source: Hum Brain Mapp. 2025 Mar 12;46(4):e70182. doi: 10.1002/hbm.70182 (PMC11897728; doi:10.1002/hbm.70182)

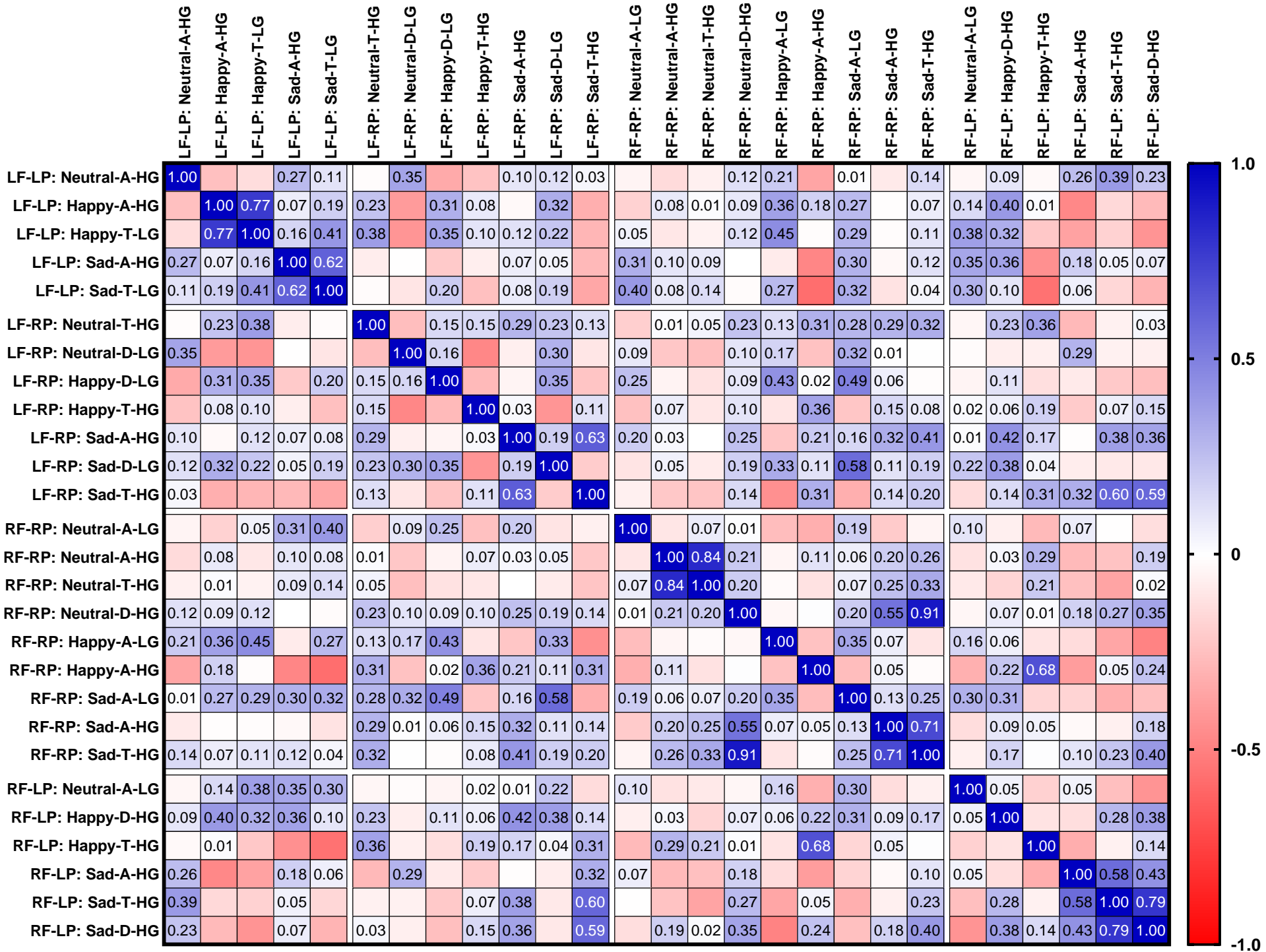

Supplement: Supplementary file 1 — Figure S1. Pearson correlation matrix for Figure 6. [file HBM-46-e70182-s001.pdf]

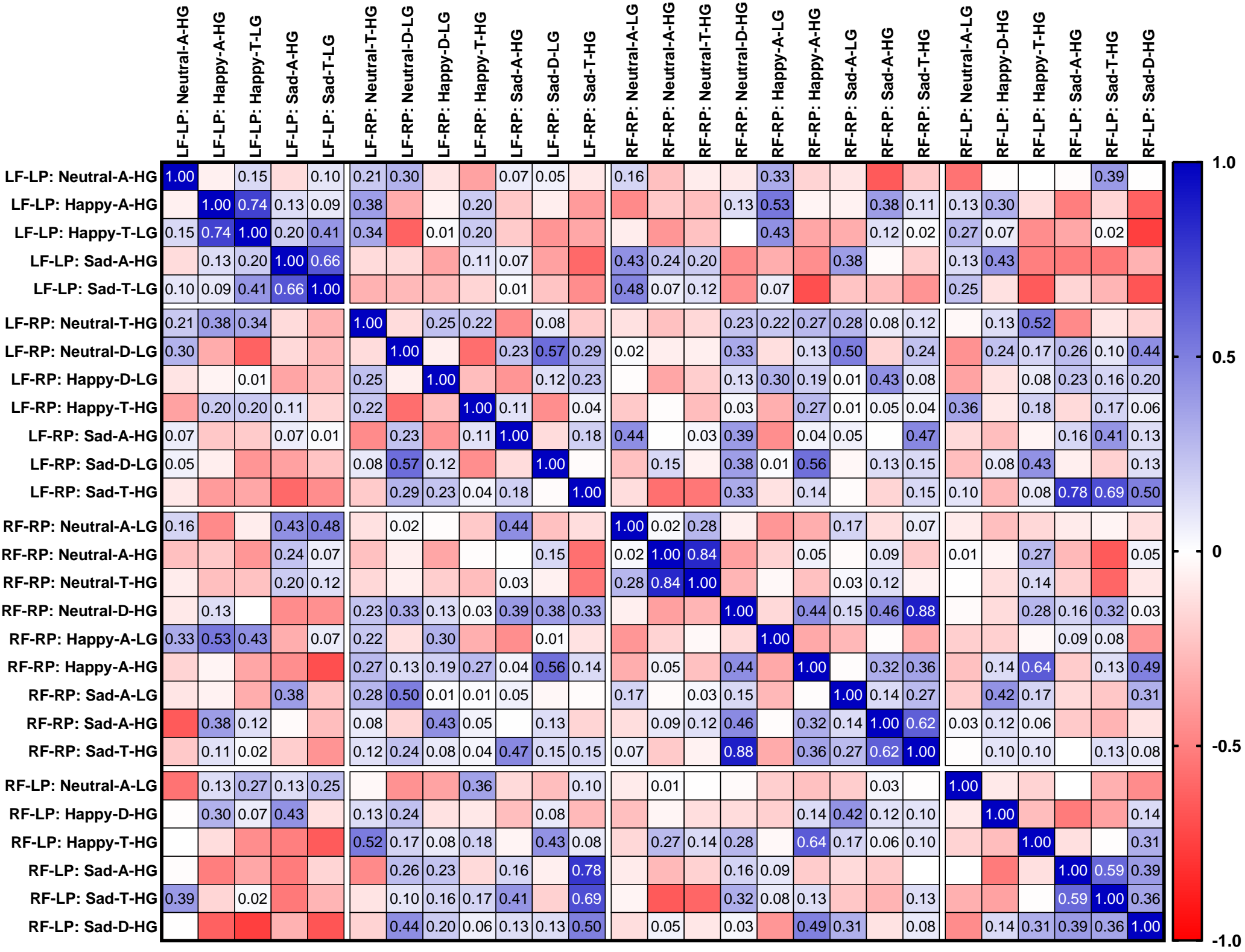

Supplement: Supplementary file 2 — Figure S2. Pearson correlation matrix for Figure 7a. [file HBM-46-e70182-s002.pdf]

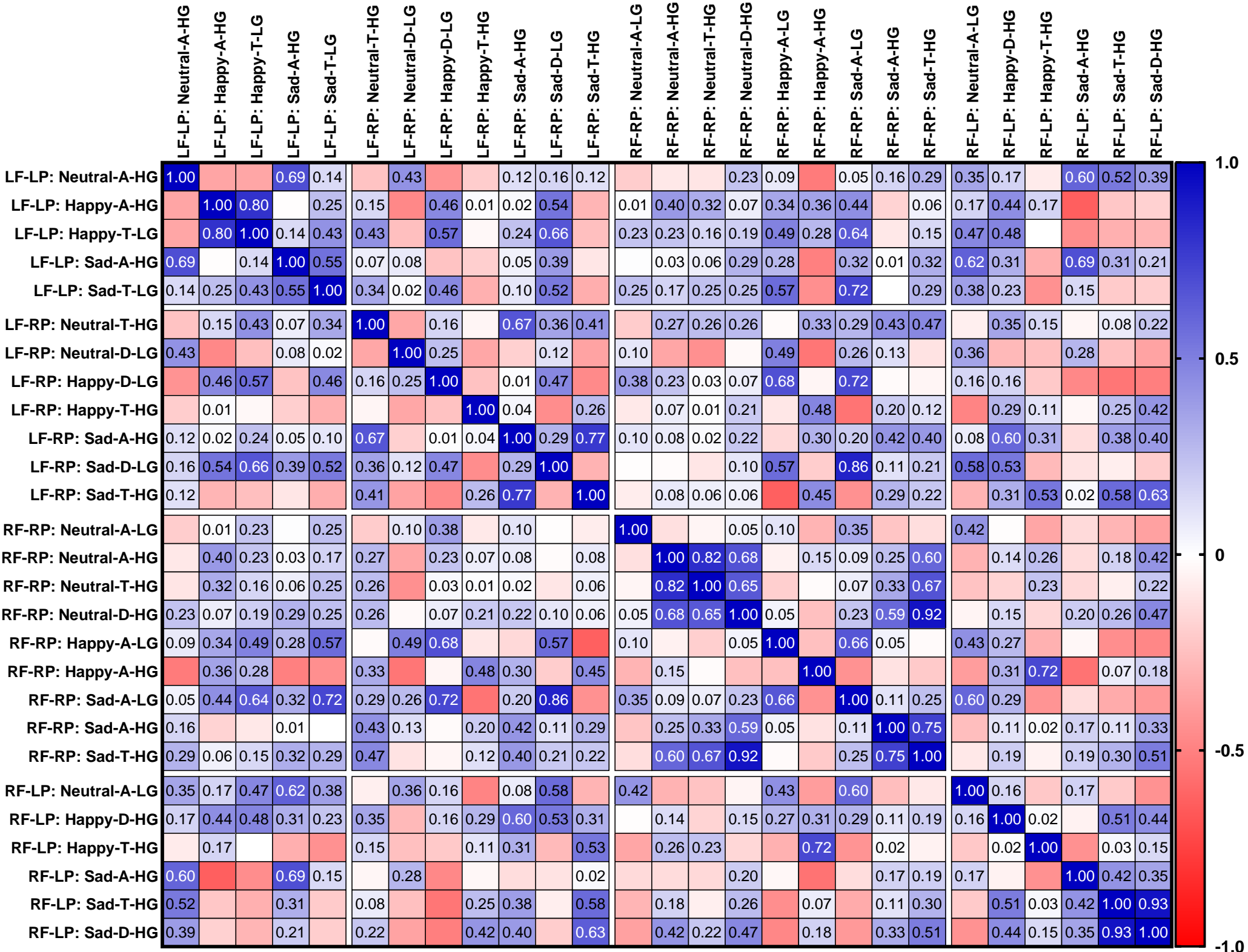

Supplement: Supplementary file 3 — Figure S3. Pearson correlation matrix for Figure 7b. [file HBM-46-e70182-s003.pdf]
